# Facility-based stillbirth review processes used in different countries across the world: a systematic review

**DOI:** 10.1016/j.eclinm.2023.101976

**Published:** 2023-04-27

**Authors:** Yebeen Ysabelle Boo, Uchenna Gwacham-Anisiobi, Dixa B. Thakrar, Nia Roberts, Jennifer J. Kurinczuk, Monica Lakhanpaul, Manisha Nair

**Affiliations:** a National Perinatal Epidemiology Unit, Nuffield Department of Population Health, University of Oxford, Oxford, United Kingdom; b Cancer Epidemiology Unit, Nuffield Department of Population Health, University of Oxford, Oxford, United Kingdom; c Bodleian Health Care Libraries, University of Oxford, Oxford, United Kingdom; d UCL Great Ormond Street Institute of Child Health, Faculty of Population Health Sciences, University College London, London, United Kingdom

**Keywords:** Stillbirth, Perinatal mortality, Mortality audit, Logic model, Systematic review

## Abstract

**Background:**

Facility-based stillbirth review provides opportunities to estimate incidence, evaluate causes and risk factors for stillbirths, and identify any issues related to the quality of pregnancy and childbirth care which require improvement. Our aim was to systematically review all types and methods of facility-based stillbirth review processes used in different countries across the world, to examine how stillbirth reviews in facility settings are being conducted worldwide and to identify the outcomes of implementing the reviews. Moreover, to identify facilitators and barriers influencing the implementation of the identified facility-based stillbirth reviews processes by conducting subgroup analyses.

**Methods:**

A systematic review of published literature was conducted by searching MEDLINE (OvidSP) [1946-present], EMBASE (OvidSP) [1974-present], WHO Global Index Medicus (globalindexmedicus.net), Global Health (OvidSP) [1973–2022 Week 8] and CINAHL (EBSCOHost) [1982-present] from their inception until 11 January, 2023. For unpublished or grey literature, the WHO databases, Google Scholar and ProQuest Dissertations & Theses Global were searched, as well as hand searching the reference lists of included studies. MESH terms encompassing “∗Clinical Audit”, “∗Perinatal Mortality”, “Pregnancy Complications”, and “Stillbirth” were used with Boolean operators. Studies that used a facility-based review process or any approach to evaluate care prior to stillbirth, and explained the methods used were included. Reviews and editorials were excluded. Three authors (YYB, UGA, and DBT) independently screened and extracted data, and assessed the risk of bias using an adapted JBI's Checklist for Case Series. A logic model was used to inform the narrative synthesis. The review protocol was registered with PROSPERO, CRD42022304239.

**Findings:**

A total of 68 studies from 17 high-income (HICs) and 22 low-and-middle-income countries (LMICs) met the inclusion criteria from a total of 7258 identified records. These were stillbirth reviews conducted at different levels: district, state, national, and international. Three types were identified: audit, review, and confidential enquiry, but not all desired components were included in most processes, which led to a mismatch between the description of the type and the actual method used. Routine data from hospital records was the most common data source for identifying stillbirths, and case assessment was based on stillbirth definition in 48 out of 68 studies. Hospital notes were the most common source of information about care received and causes/risk factors for stillbirth. Short-term and medium-term outcomes were reported in 14 studies, but impact of the review process on reducing stillbirth, which is more difficult to establish, was not reported in any study. Facilitators and barriers in implementing a successful stillbirth review process identified from 14 studies focused on three main themes: resources, expertise, and commitment.

**Interpretation:**

This systematic review's findings identified that there is a need for clear guidelines on how to measure the impact of implementation of changes based on outputs of stillbirth reviews and methods to enable effective dissemination of learning points in the future and promoting them through training platforms. In addition, there is a need to develop and adopt a universal definition of stillbirth to facilitate meaningful comparison of stillbirth rates between regions. The key limitation of this review is that while using a logic model for narrative synthesis was deemed most appropriate for this study, sequence of implementing a stillbirth review in the real world is not linear, and assumptions are often not met. Therefore, the logic model proposed in this study should be interpreted with flexibility when designing a stillbirth review process. The generated learnings from the stillbirth review processes inform the action plans and allow facilities to consider where the changes should happen to improve the quality of care in the facilities, enabling positive short-term and medium-term outcomes.

**Funding:**

10.13039/100010348Kellogg College, University of Oxford, 10.13039/501100014748Clarendon Fund, University of Oxford, Nuffield Department of Population Health, 10.13039/501100000769University of Oxford and 10.13039/501100000265Medical Research Council (MRC).

**Research in context**

**Evidence before this study**

Stillbirth review at the facility level is especially critical, as it provides opportunities to review the incidence rate, causes and risk factors for stillbirths, as well as identify any issues related to the quality of pregnancy and childbirth care at the hospital. The risk factors and quality of care gaps identified may inform the implementation of evidence-based interventions for reducing facility-based stillbirth in the future. However, we could find no evidence of a systematic review that critically appraised and synthesised the methods used for facility-based stillbirth review process across the world, in detail, to date when following databases were searched from their inception until 11 January, 2023: MEDLINE (OvidSP) [1946-present], EMBASE (OvidSP) [1974-present], WHO Global Index Medicus (globalindexmedicus.net), Global Health (OvidSP) [1973–2022 Week 8] and CINAHL (EBSCOHost) [1982-present]. We also could not find any unpublished or grey literature that met our inclusion criteria from the World Health Organization (WHO) databases, Google Scholar and ProQuest Dissertations & Theses Global. The inclusion criteria for our search was any systematic reviews that reviewed all types and methods of facility-based stillbirth review processes used in different countries across the world and any systematic reviews that only reviewed types and methods of facility-based stillbirth review processes used in particular country settings or certain income levels only were excluded. MESH terms encompassing “∗Clinical Audit”, “∗Perinatal Mortality”, “Pregnancy Complications”, and “Stillbirth” were used with Boolean operators for our search and there were no language or geographical restrictions.

**Added value of this study**

This systematic review is the first that we know of, to systematically review all types and methods of facility-based stillbirth review processes used in different countries across the world. It uses a robust methodology by including independent reviewers for screening, extracting and quality assessment. The systematic review demonstrated that stillbirth review processes are not standardised in many countries at a local/national level, despite their known importance in identifying gaps in care. However, we found that the learnings from the stillbirth review processes were able to inform action plans and allowed facilities to consider where the changes should be made to improve the quality of care in the facilities, thus enabling positive short-term and medium-term outcomes. Additionally, we identified facilitators and barriers influencing the implementation of the identified facility-based stillbirth review processes from the included studies.

**Implications of all the available evidence**

This systematic review identified different types and methods of facility-based stillbirth review processes implemented around the world and a logic model was built from the extracted evidence to describe what a successful stillbirth review process might encompass. The logic model could be used as a guide or contextually adapted by health facilities to improve their stillbirth review process. Since most stillbirths (98%) occur in low-and-middle-income countries (LMICs), there is an urgent need to introduce standardised stillbirth review processes in more LMICs, while considering the facilitators and barriers, resources required, training and support needs.

## Introduction

Stillbirth reviews were started in the United Kingdom (UK) as part of the Confidential Enquiry into Stillbirths and Deaths in Infancy (CESDI) in 1992, which was an expansion of the already established and long running Confidential Enquiry into Maternal Deaths (CEMD).^1^ A stillbirth is a baby born with no signs of life after a given threshold, usually related to the gestational age or birthweight. The rationale for the establishment of stillbirth reviews in the UK was to identify ways to prevent stillbirths and reduce stillbirth rates in the country. In 2016, the World Health Organization (WHO) released a guide: Making Every Baby Count: Audit and review of stillbirths and neonatal deaths, to encourage countries to implement stillbirth reviews to identify underlying reasons why stillbirths occurred and what can be learnt to prevent stillbirths in the future.^2^

The fundamental aim of a stillbirth review is to “support objective, robust and standardised review to provide answers for bereaved parents about why their baby died”^3^ and to “ensure local and national learning to improve care and ultimately prevent future deaths.”^3^ Stillbirth review at the facility level is especially critical, as it provides opportunities to review the incidence rate, causes and risk factors for stillbirths, as well as identify any issues related to the quality of pregnancy and childbirth care at the hospital. The identified risk factors and issues with quality of care can then be used to inform the implementation of evidence-based interventions for reducing facility-based stillbirth in the future. However, we could find no evidence of a systematic review that critically appraised and synthesised the methods used for facility-based stillbirth review process across the world, in detail, to date.

Our aim was to systematically review all types and methods of facility-based stillbirth review processes used in different countries across the world, both high-income countries (HICs) and low-and-middle-income countries (LMICs). The primary objective was to examine how stillbirth reviews in facility settings are being conducted worldwide and to identify the outcomes of implementing the reviews. The secondary objective was to identify facilitators and barriers influencing the implementation of the identified facility-based stillbirth reviews processes by conducting subgroup analyses.

## Methods

### Search strategy and selection criteria

The protocol for this systematic review was developed prospectively and was registered before any stage of the systematic review was completed. The Unique Identification number of PROSPERO is CRD42022304239.^4^ Preferred Reporting Items for Systematic Reviews and Meta-Analyses (PRISMA) guideline was followed to report our findings.

The following electronic bibliographic databases were searched: MEDLINE (OvidSP) [1946-present], EMBASE (OvidSP) [1974-present], WHO Global Index Medicus (globalindexmedicus.net), Global Health (OvidSP) [1973–2022 Week 8] and CINAHL (EBSCOHost) [1982-present]. For unpublished or grey literature, the WHO databases (who.int), Google Scholar (scholar.google.com) and ProQuest Dissertations & Theses Global (proquest.com) were searched, as well as hand searching the reference lists of included studies. MESH terms encompassing “∗Clinical Audit”, “∗Perinatal Mortality”, “Pregnancy Complications”, and “Stillbirth” were used with Boolean operators. The searches employed sensitive, topic-based strategies designed for each database, and used terms related to or describing “stillbirth review” using relevant Cochrane review's search terms as a guide^5^ (see Supplementary material: Appendix 1). The search dates were from the inception of the databases to the 10th of December 2021 and were updated every month to identify any further studies for inclusion. The final search was conducted on the 11th of January 2023, and the references were imported into Covidence software.^6^

The screening was conducted as a two-stage process. Firstly, all titles and abstracts were screened based on inclusion and exclusion criteria followed by a full-text screening. Both stages were carried out independently by three reviewers; the first reviewer (YYB) reviewed all the articles and two other reviewers (UGA and DBT) reviewed 50% each. In both stages, if there was a disagreement, the three reviewers worked to resolve by consensus, and if it was not resolved after the consensus meeting, a senior supervisor (MN) was consulted. Cohen's kappa was used to calculate the level of agreement among reviewers.

### Inclusion and exclusion criteria

The systematic review included studies that used a facility-based review process or a review tool to investigate stillbirths (antepartum and/or intrapartum) and explained the methods used. This means, any stillbirth review process in a facility-based setting, whether as a subgroup of perinatal death review or a standalone process to investigate stillbirths, and any other type of review processes such as audit, review/meeting, confidential enquiries or any combination of these were eligible for inclusion. A stillbirth review is more extensive than stillbirth surveillance, and investigates the quality of care, attempts to identify avoidable factors (resulting from suboptimal care), and/or modifiable factors in addition to information about identification of stillbirth, pregnancy progress, and care, and details of the labour and birth. There were no restrictions for age range, ethnicity, or any health status information in terms of the women who were included in the reviews. There were no language or geographical restrictions and all types of studies were considered except systematic reviews, case reports and editorials.

### Data extraction and analysis

A data extraction template was developed including four main sections: general information, the facility-based stillbirth review process, outputs and outcomes, and facilitators and barriers to implementation of the stillbirth review (see Supplementary material: Appendix 2). Data were extracted directly into Covidence software by the first reviewer (YYB), and two other reviewers independently reviewed this information (UGA and DBT).

Several tools for risk of bias assessment and different types of frameworks for narrative synthesis were considered and piloted before finalising the most applicable methods to appraise the included studies. The quality assessment of individual studies was conducted independently by three reviewers (YYB, UGA and DBT) using an adapted version of “The Joanna Briggs Institute Critical Appraisal tools for use in JBI Systematic Reviews: Checklist for Case Series”^7^ (see Supplementary material: Appendix 3).

Using the existing template developed by Taylor-Powell et al.*,*^8^ a theoretical logic model was developed *de novo* to present a narrative synthesis of the findings in a useable format of all studies included in the review. The logic model was finalised when a consensus was reached between all co-authors via multiple iterations following several group discussions. The types of stillbirth reviews were categorised by critically appraising the methods described for the review process and comparing them against standard guidance which were informed by an integrative literature review article by Helps et al. (2020), outlining the usual characteristics of perinatal audits, local reviews and confidential enquiries.^9^ Helps et al. state that audit involves data collection, case assessment of stillbirth, implementation of changes based on outputs and re-evaluation, whereas, a review includes components of “a collaboration between different specialities in obstetrics, midwifery, neonatology and pathology i.e. a multidisciplinary team”, to examine reasons for cases for suboptimal care and/or avoidable factors, and dissemination of key findings and learning points to all relevant clinical staff.^9^ In addition, a confidential inquiry consists of all the aspects of a review but is distinguished by cases being identified via “an anonymous review” process.^9^ This is different from the usual standards of anonymity; reviewers receive case notes with no prior knowledge or links and, therefore, can offer a fresh pair of critical but fair eyes.

### Role of the funding source

The funder of the study had no role in study design, data collection, data analysis, data interpretation, or writing of the report. All researchers were independent of the funders and all authors had access to the data and accept responsibility for the decision to submit for publication.

## Results

**Fig. 1:**
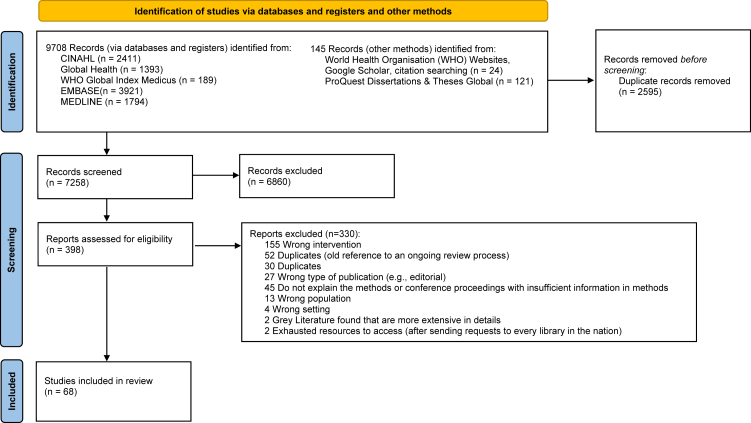
PRISMA flowchart of study selection. *From:* Page MJ, McKenzie JE, Bossuyt PM, Boutron I, Hoffmann TC, Mulrow CD, et al. The PRISMA 2020 statement: an updated guideline for reporting systematic reviews. BMJ 2021;372:n71. https://doi.org/10.1136/bmj.n71. For more information, visit: http://www.prisma-statement.org/

We identified 7258 titles and abstracts eligible for the first stage of screening. After excluding 6860 records, which did not meet the inclusion criteria, 398 full texts were screened in the second stage. After completing the screening process, a total of 68 studies met the inclusion criteria and were finally included in the systematic review (Fig. 1). If there were studies that used the same methods and reports produced annually, the most recent report was included. The inter-rater reliability comparing the percent agreement between reviewers during the title and abstract screening was 0.8 (Cohen's Kappa statistics = 0.11) and full-text screening was 0.9 (Cohen's Kappa statistics = 0.35).

**Fig. 2:**
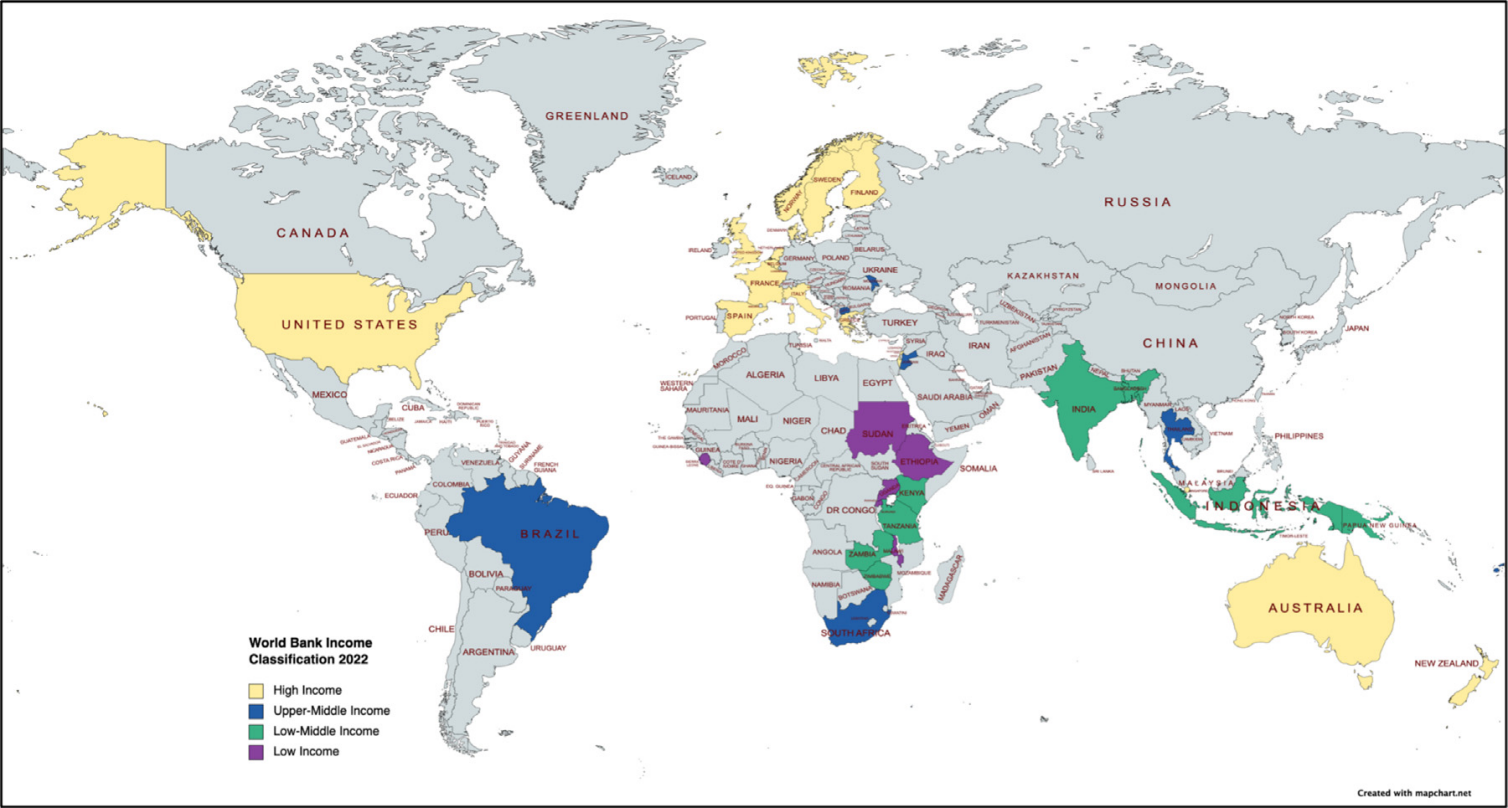
The geographical distribution of countries of included studies using the World Bank income classification 2022.

Table 1 summarises the country and its income category as per the World Bank Income Classification (WBIC), time-period of the study, and the level (as described below) at which the facility-based stillbirth reviews were conducted for the 68 included studies. The 68 studies were from 39 countries (Fig. 2). When categorising the countries using the WBIC 2022, 39 studies were from 17 HICs and 29 were from 22 LMICs.^76^ There was variation among the studies in the inclusion of these characteristics, and in the ranges included, regardless of the type of country involved.

**Table 1:** General information about the included studies.

First author, publication year	Country	Level of review	Duration
**High-income countries**			
Flenady, 2021^10^	Australia	D	1 Jan 2018–1 Dec 2018
Richardus, 2003^11^	Belgium; Denmark; Finland; Greece; Netherlands; Norway; Spain; United Kingdom	I	1993–1998
Andersen, 1991^12^	Denmark	D	1985–1986
Westergaard, 1997^13^	Denmark; Sweden	I	1991
Sauvegrain, 2020^14^	France	D	1 Jan-31 Dec 2014
de Caunes, 1990^15^	Guadeloupe^a^	N	Nov 1983–Dec 1985
Furst, 1989^16^	Israel	L	Oct 1987–Feb 1988
D'Aloja, 2021^17^	Italy	L; D; N	1 Jul 2017–30 Jun 2019
Alderliesten, 2008^18^	Netherlands	D	1999
Dekker, 2003^19^	Netherlands	L	1986–1995
Eskes, 1993^20^	Netherlands	L	1969–1983
van Diem, 2010^21^	Netherlands	Oˆ	Nov 2004–May 2005
van Diem, 2012^22^	Netherlands	D	Sep 2007–Mar 2010
Wolleswinkel-van den Bosch, 2002^23^	Netherlands	D	1996–1997
Eskes, 2014^24^	Netherlands	N	2010-present
PMMRC, 2021^25^	New Zealand	N	2007-present
Berge, 1991^26^	Norway	L	1976–89
Bjellmo, 2019^27^	Norway	N	1999–2015
Fossen, 1999^28^	Norway	D	1989–1997
Han, 2018^29^	Singapore	L	Jan 2004–Dec 2008
De la Puente, 2002^30^	Spain	L	1997–1998
Miranda, 1996^31^	Spain	L	1979–1992
Eksmyr, 1986^32^	Sweden	D	1973–1978
Essén, 2002^33^	Sweden	N	1990–1996
Sterpu, 2020^34^	Sweden	D	2017
Chepkin, 2019^3^	United Kingdom	L	Feb 2018-present
Cross-Sudworth, 2015^35^	United Kingdom	L	2008
Draper, 2017^36^	United Kingdom	N	Nov 2016–May 2017
Hundley, 2001^37^	United Kingdom	D	Not stated (re-review of the review that was conducted during the Aberdeen Trial in 1992–1993)
Maternal and Child Health Research Consortium, 1999^38^	United Kingdom	N	The Confidential Enquiry into Stillbirth and Deaths in Infancy (CESDI) from 1993–2002; the Confidential Enquiries into Maternal and Child Health (CEMACH) from 2003–2008; Centre for Maternal and Child Enquiries (CMACE) from 2009–2011
Mersey Region Working Party on Perinatal Mortality, 1982^39^	United Kingdom	D	1979
Tan, 1999^40^	United Kingdom	D	1991
Tang, 2011^41^	United Kingdom	L	2004–2009
Bausch, 1996^42^	United States	D	1992
Harper, 1977^43^	United States	D	1973
Kieltyka, 2012^44^	United States	D	From 2001
Moawad, 1990^45^	United States	D	1983–1987
The National Center for Fatality Review and Prevention, 2021^46^	United States	N	Late 1980s- present
Vallejo, 1991^47^	United States	D	1988
**Upper-middle income countries**			
Amaral, 2011^48^	Brazil	D	Oct–Dec 2005
Raman, 2015^49^	Fiji	L	2011–12
Alyahya, 2021^50^	Jordan	L	1 Aug 2019–1 Feb 2020
Stratulat, 2014^51^	Moldova	N	2006–2010
Copenhagen: WHO Regional Office for Europe, 2020^52^	North Macedonia	N	2019
Govender, 2017^53^	South Africa	L	1 Apr 2014–31 Mar 2015
Pattinson, 1995^54^	South Africa	D	Aug 1991–Jul 1992
Rhoda, 2014^55^	South Africa	N	From the early 1990s, but was not a truly national programme until 2012.
Ward, 1995^56^	South Africa	L	1 Jan 1991–31 Dec 1992
Wilkinson, 1997^57^	South Africa	D	May 1991–Dec 1995
Mo-suwan, 2009^58^	Thailand	D	15 Oct 2000–19 Mar 2002
**Low income and low-and-middle income countries**			
Demise, 2015^59^	Ethiopia	L	Jun–Nov 2012
Musafili, 2017^60^	Rwanda	L	Jul 2012–May 2013
El Amin, 2002^61^	Sudan	L	May–Aug 2000
Kirabira, 2020^62^	Uganda	L	From 2008
Biswas, 2015^63^	Bangladesh	D	2010–2011
Bhatt, 1989^64^	India	L	Initiated from 1965
Sharma, 2022^65^	India	L	Dec 2018–Nov 2019
Supratikto, 2002^66^	Indonesia	D	From 1994
Bandali, 2019^67^	Kenya	O^+^	From 2014
Omwodo, 2020^68^	Kenya	L	1 May 2017–31 Aug 2018
Aminu, 2017^69^	Kenya; Malawi; Sierra Leone; Zimbabwe	L	Jan–Sep 2015
Vallely, 2020^77^	Papua New Guinea	D	Jul 2017–Jan 2020
Hinderaker, 2003^70^	Tanzania	D	Not stated
Maaløe, 2016^71^	Tanzania	L	1 Oct 2014–31 Jan 2015
Mbaruku, 2009^72^	Tanzania	L	Jul 2002–Jul 2004
Mdoe, 2022^73^	Tanzania	L	Jan 2019–May 2020
Wilkins, 2015^74^	Timor-Leste	L	Nov 2009–Dec 2010
Kasengele, 2017^75^	Zambia	L	2012–2014

L: local/hospital(s), D: district/state, N: national, I: international.

O+ It is implemented nationally. However, this paper focuses on Bungoma country as it is the only country in Kenya that has reviewed more than 50% of perinatal deaths in 2017.

Oˆ Included three regions, therefore between D and N level.

a Guadeloupe was included as a high-income country as it is a French overseas region.

The level of review was categorised based on the place, and/or the region where the review was conducted. They could include national guidance and tools, but if the review described in the study was conducted at a regional level, it was considered a regional level review. However, if the study conducted its review in different hospitals/regions in more than one country, the study was considered an international review.

Twenty-seven studies were stillbirth reviews conducted at a local or hospital level (not in the entire district), 25 were conducted at a district or state level, 11 were conducted at a national level and two were conducted at an international level. Three further studies that could not be categorised, as one study was conducted at a national level, however, the study focused on a county called Bungoma as it was the only county in the country (Kenya) that had reviewed more than 50% of stillbirths in 2017, whereas other counties had a very low review rate.^67^ Another study was conducted at all three levels (district, state, and national), as their process was a three-tiered system.^17^ Lastly, one study was a feasibility study to understand if the audit could be performed at a national level and involved three regions in a country.^21^

There were three types of stillbirth reviews as described by the authors of the included studies – audit, review, and confidential enquiry. It is important to note that some studies may have used the word audit and review interchangeably, though they have not explicitly stated that they used the term interchangeably in their studies. Therefore, the types were categorised by critically appraising the methods described for the review process and comparing them against standard guidance which were informed by an integrative literature review by Helps et al. (2020) as described in the methods section. Table 2 presents a comparison of the author definition of type of review in the included studies and the salient features of the usual processes as discussed above, and the results are shown as count and percentage of included studies for which the author definition matched with the salient features described by Helps et al.. Out of 41 studies that defined their review method as an “audit”, most included a component of data collection (95%), while re-evaluation (i.e., a continuous cycle) was the most lacking aspect (27%). Many, however, also had components of a review and confidential enquiry (Table 2 and Supplementary material: Appendix 4). A total of 28 studies that defined their review method as a “review” or “confidential enquiry” also showed similar patterns to studies that defined their review method as an “audit”; not all studies contained the desired components of a review/confidential enquiry.

**Table 2:** Count and percentage of studies that had the component of relevant stillbirth review depending on the type (audit, review, and confidential enquiry).

Salient features of the usual process as described by Helps et al. (2020)	As described by authors in the included studies		
	Audit (n = 41)	Review (n = 28)	Confidential enquiry (n = 14)
Audit component 1: data collection	39 (95%)	28 (100%)	14 (100%)
Audit component 2: case assessment of stillbirth	33 (81%)	24 (86%)	13 (93%)
Audit component 3: implementation of changes based on outputs	12 (29%)	9 (32%)	3 (21%)
Audit component 4: re-evaluation (i.e., a continuous cycle)	11 (27%)	10 (36%)	3 (21%)
Review component 1: a collaboration between different specialities in obstetrics, midwifery, and pathology (i.e., the multidisciplinary team)	23 (56%)	21 (75%)	13 (93%)
Review component 2: examine reasons (what, when, who) for cases for suboptimal care and/or avoidable factors	33 (81%)	22 (79%)	12 (86%)
Review component 3: dissemination of key findings and learning points to all relevant clinical staff	13 (32%)	11 (39%)	5 (36%)
Confidential enquiry: anonymous in addition to the above three components of a review process	5 (12%)	12 (43%)	11 (79%)

The included studies had one or more of the following aims when conducting a stillbirth review: to prevent stillbirth, to improve all aspects of care by making adequate and informed recommendation guided by clear local/national/international criteria or practices^3^^,^^15^^,^^17^^,^^25^^,^^36^^,^^39^^,^^43^^,^^46^^,^^51^^,^^52^^,^^55^, ^56^, ^57^^,^^64^^,^^65^^,^^69^^,^^73^^,^^75^; to analyse cause/s of stillbirth and/or any suboptimal care during the pregnancy^3^^,^^10^^,^^11^^,^^15^^,^^16^^,^^19^, ^20^, ^21^, ^22^, ^23^, ^24^^,^^26^^,^^28^, ^29^, ^30^, ^31^, ^32^^,^^36^, ^37^, ^38^, ^39^, ^40^, ^41^, ^42^, ^43^^,^^45^^,^^47^^,^^50^^,^^53^, ^54^^,^^58^^,^^59^^,^^61^^,^^66^, ^67^, ^68^, ^69^, ^70^, ^71^, ^72^, ^73^, ^74^^,^^77^; to evaluate the current review process or tool implemented and what the facilitators or barriers were^18^^,^^32^^,^^34^^,^^44^^,^^48^^,^^49^^,^^53^^,^^62^^,^^63^; and to compare if there were differences in stillbirth rate between groups of pregnant women that occurred due to systematic differences in quality of care.^12^, ^13^, ^14^^,^^27^^,^^33^^,^^35^

For 29 out of 68 studies (43%), Government agencies (15 studies^3^^,^^14^^,^^17^^,^^18^^,^^24^^,^^25^^,^^30^^,^^32^^,^^34^, ^35^, ^36^^,^^38^^,^^39^^,^^72^^,^^75^) or other funding bodies such as non-governmental organisations (9 studies^19^^,^^20^^,^^22^^,^^23^^,^^51^^,^^62^^,^^67^^,^^69^^,^^73^), universities or professional associations (2 studies^26^^,^^47^), and research grants (3 studies^10^^,^^11^^,^^77^) were solely responsible for commissioning the review process. Fifteen studies^12^^,^^15^^,^^27^^,^^33^^,^^43^^,^^46^^,^^48^^,^^50^^,^^58^^,^^60^^,^^63^^,^^66^^,^^70^^,^^71^ out of 68 received funding from two or more organisations (22%), but 24 out of the 68 studies (35%) received no funding or did not list any funding information for the stillbirth review process.

Routine data was used as at least one of the data sources to identify eligible stillbirths for review in 44 out of 68 included studies (65%).^10^, ^11^, ^12^^,^^16^^,^^19^^,^^22^, ^23^, ^24^, ^25^, ^26^, ^27^, ^28^, ^29^, ^30^, ^31^, ^32^, ^33^, ^34^^,^^38^^,^^42^, ^43^, ^44^, ^45^, ^46^, ^47^, ^48^^,^^50^^,^^52^^,^^55^^,^^57^^,^^59^^,^^61^, ^62^, ^63^, ^64^, ^65^^,^^67^^,^^69^^,^^71^, ^72^, ^73^, ^74^, ^75^^,^^77^ Routine data included birth or death registry, hospital records, social care organisations' records, and obstetric records. Four studies (6%) used interviews with care providers as a supplementary source of data, in addition to routine records to identify stillbirths for review.^22^^,^^30^^,^^63^^,^^72^ Sixteen studies (24%) implemented a specific data identification system to build a stillbirth database only for the purposes of the review or to enable the review to be started.^3^^,^^14^^,^^15^^,^^17^^,^^18^^,^^24^^,^^25^^,^^36^, ^37^, ^38^, ^39^^,^^53^^,^^58^^,^^66^^,^^67^^,^^70^ For example, they requested care providers to fill in a form within a reasonable time after the death in the facility and report through an online surveillance system or via phone call/fax to notify the review team to enable a review to be started.^3^^,^^17^^,^^18^^,^^38^^,^^39^^,^^66^^,^^67^ Some studies used an existing specific database that was not built for the purpose of the stillbirth review only, but to support research on how to reduce stillbirth. For example, a study utilised a prospective cohort of antenatal attendees’ data to identify stillbirth within the cohort for review.^58^ Twelve studies (18%) did not state what data source they used to identify stillbirths.^13^^,^^20^^,^^35^^,^^40^^,^^49^^,^^51^^,^^54^^,^^56^^,^^60^^,^^68^ Please see Supplementary material: Appendix 5 for the detailed lists of sources of data used for each study.

There is no universally accepted definition of stillbirth and the definition tends to vary between countries and within countries,^78^ an issue also observed in the included studies. Out of the 68 studies, 20 did not list any definition of stillbirth used for case assessment, 29 studies used only one criterion (either gestational age or birthweight) to define stillbirths for case assessment, and three studies used three criteria (gestational age, birthweight, and crown-heel length) (Table 3). Sometimes case assessment was limited to particular interests as the review may focus on one aspect of stillbirth; for example, four studies^13^^,^^36^^,^^51^^,^^72^ only focused on intrapartum stillbirth, two studies^10^^,^^24^ only focused on late gestational stillbirth, and one study^40^ only included stillbirths with birthweight above 2500 g considering that these were most likely to be “influenced by obstetric care and management”. Excluding seven studies that only focused on a particular type of stillbirth, the minimum gestation age used ranged between 20 and 28 weeks. The minimum birthweight used ranged between 400 g and 1000 g, and minimum crown-heel length used ranged between 25 and 35 cm. When dividing this range by country's income classification, studies that were conducted in LMICs had a narrower gestation age range of 22–28 weeks, a narrower birthweight range of 500–1000 g and crown-heel length of 35 cm. Compared to HICs, in LMICs the minimum gestational age was higher by two weeks, the minimum birthweight was higher by 100 g, and the minimum crown-heel length was also higher by 10 cm. In addition, six studies^13^^,^^17^^,^^36^^,^^38^^,^^68^^,^^75^ used additional definitions to classify antepartum/macerated and intrapartum/fresh stillbirths; all six studies used the “onset of labour” or “onset of care in labour” as a criterion to differentiate the two types of stillbirths.

**Table 3:** Definition (case assessment of stillbirth) used in included studies.

First author and year	Country	Age of gestation (weeks)	Birthweight (g)	Crown-heel Length (cm)
**High-income countries**				
Flenady, 2021^10^	Australia	≥34^b^	N/A	
Richardus, 2003^11^	Belgium; Denmark; Finland; Greece; Netherlands; Norway; Spain; United Kingdom	≥28	N/A	
Andersen, 1991^12^	Denmark	≥28	N/A	
Westergaard, 1997^13^	Denmark; Sweden	>28^a^	N/A	
Sauvegrain, 2020^14^	France	≥22	N/A	
de Caunes, 1990^15^	Guadeloupe	≥22	N/A	
Furst, 1989^16^	Israel	N/A	N/A	
D'Aloja, 2021^17^	Italy	≥28	N/A	
Alderliesten, 2008^18^	Netherlands	N/A	N/A	
Dekker, 2003^19^	Netherlands	>24	≥500	
Eskes, 1993^20^	Netherlands	N/A	>500	
van Diem, 2010^21^	Netherlands	N/A	N/A	
van Diem, 2012^22^	Netherlands	>22	>500	≥25
Wolleswinkel-van den Bosch, 2002^23^	Netherlands	≥24	N/A	
Eskes, 2014^24^	Netherlands	≥37^b^	N/A	
PMMRC, 2021^25^	New Zealand	≥20	≥400	
Berge, 1991^26^	Norway	≥24	N/A	
Bjellmo, 2019^27^	Norway	N/A	N/A	
Fossen, 1999^28^	Norway	≥22	N/A	
Han, 2018^29^	Singapore	≥28	≥500	
De la Puente, 2002^30^	Spain	≥22	>500	
Miranda, 1996^31^	Spain	N/A	≥1000	
Eksmyr, 1986^32^	Sweden	N/A	N/A	
Essén, 2002^33^	Sweden	N/A	N/A	
Sterpu, 2020^34^	Sweden	≥22 + 0	N/A	
Chepkin, 2019^3^	United Kingdom	≥22 + 0	>500	
Cross-Sudworth, 2015^35^	United Kingdom	≥24	N/A	
Draper, 2017^36^	United Kingdom	≥37^a^	N/A	
Hundley, 2001^37^	United Kingdom	N/A	N/A	
Maternal and Child Health Research Consortium, 1999^38^	United Kingdom	>24	N/A	
Mersey Region Working Party on Perinatal Mortality, 1982^39^	United Kingdom	N/A	N/A	
Tan, 1999^40^	United Kingdom	N/A	≥2500^c^	
Tang, 2011^41^	United Kingdom	N/A	N/A	
Bausch, 1996^42^	United States	N/A	N/A	
Harper, 1977^43^	United States	N/A	N/A	
Kieltyka, 2012^44^	United States	≥24	N/A	
Moawad, 1990^45^	United States	N/A	≥501	
The National Center for Fatality Review and Prevention, 2021^46^	United States	N/A	N/A	
Vallejo, 1991^47^	United States	≥20	≥500	
**Low-and-middle income countries**				
Biswas, 2015^63^	Bangladesh	N/A	N/A	
Amaral, 2011^48^	Brazil	N/A	≥500	
Demise, 2015^59^	Ethiopia	≥28	≥1000	
Raman, 2015^49^	Fiji	N/A	N/A	
Bhatt, 1989^64^	India	N/A	N/A	
Sharma, 2022^65^	India	≥28	≥1000	
Supratikto, 2002^66^	Indonesia	N/A	N/A	
Alyahya, 2021^50^	Jordan	≥24	N/A	
Bandali, 2019^67^	Kenya	N/A	N/A	
Omwodo, 2020^68^	Kenya	≥22	≥500	
Aminu, 2017^69^	Kenya; Malawi; Sierra Leone; Zimbabwe	≥28	≥1000	≥35
Stratulat, 2014^51^	Moldova	>37^a^	≥2500^a^	
Copenhagen: WHO Regional Office for Europe, 2020^52^	North Macedonia	≥22	≥500	
Vallely, 2020^77^	Papua New Guinea	N/A	N/A	
Musafili, 2017^60^	Rwanda	≥22	≥500	
Govender, 2017^53^	South Africa	N/A	≥500	
Pattinson, 1995^54^	South Africa	N/A	>1000	
Rhoda, 2014^55^	South Africa	N/A	N/A	
Ward, 1995^56^	South Africa	>28	≥1000	
Wilkinson, 1997^57^	South Africa	N/A	N/A	
El Amin, 2002^61^	Sudan	N/A	≥500	
Hinderaker, 2003^70^	Tanzania	≥28	N/A	
Maaløe, 2016^71^	Tanzania	N/A	≥1000	
Mbaruku, 2009^72^	Tanzania	N/A	≥2000^a^	
Mdoe, 2022^73^	Tanzania	≥28	N/A	
Mo-suwan, 2009^58^	Thailand	≥28–40	N/A	
Wilkins, 2015^74^	Timor-Leste	≥22	≥500	
Kirabira, 2020^62^	Uganda	≥28	≥1000	≥35
Kasengele, 2017^75^	Zambia	N/A	N/A	

a Intrapartum stillbirth only.

b Late gestational stillbirth only.

c Stillbirth most likely to be influenced by obstetric care and management.

Abstracting or directly reviewing data from already available data (e.g. hospital notes, social care data, death registry, theatre records) was the most common method of gathering data to facilitate a stillbirth review. This means there was no interview or further additional information sought; 36 out of 68 studies (53%) used this method for data collection.^12^^,^^13^^,^^18^^,^^19^^,^^21^^,^^23^^,^^24^^,^^27^, ^28^, ^29^, ^30^, ^31^, ^32^, ^33^, ^34^^,^^36^, ^37^, ^38^^,^^42^, ^43^, ^44^, ^45^^,^^47^^,^^48^^,^^52^^,^^55^^,^^56^^,^^61^^,^^67^^,^^71^^,^^73^^,^^74^^,^^75^^,^^77^ Twenty-six studies (38%) collected additional data for the purpose of the review^3^^,^^10^^,^^11^^,^^14^, ^15^, ^16^, ^17^^,^^25^^,^^35^^,^^39^^,^^41^^,^^46^^,^^49^^,^^50^^,^^57^, ^58^, ^59^, ^60^^,^^62^, ^63^, ^64^, ^65^, ^66^^,^^69^^,^^70^^,^^72^; for example, by conducting interviews with mothers, health care providers, and family members, by requesting contextual background information about the facilities and mothers, and by carrying out additional tests (e.g. macroscopically examining placenta and cords) that would otherwise not be done. Six studies (9%) did not state how they collected data to facilitate the stillbirth review process.^20^^,^^26^^,^^40^^,^^51^^,^^54^^,^^68^ Please see Supplementary material: Appendix 5 for the detailed data collection methods used for each study.

In addition to the components listed in Table 2, there were other notable components of stillbirth review that were observed in the included studies. Please see Supplementary material: Appendix 5 for the detailed review methods/tools used for each study. These included questions related to: reviewers being trained in the review process^18^^,^^24^^,^^50^; data needed for the review being delivered to the reviewers prior to the review process to help with their preparation^12^^,^^18^^,^^36^^,^^61^^,^^52^; structured forms or checklists to fill in during the review^3^^,^^17^^,^^18^^,^^24^^,^^37^^,^^49^, ^50^, ^51^, ^52^, ^53^^,^^66^, ^67^, ^68^, ^69^^,^^71^^,^^73^ versus discussion oriented process^64^; no review meetings (chart review only)^22^^,^^26^^,^^29^^,^^41^^,^^46^^,^^49^^,^^69^^,^^73^ versus two stage review process; individual judgement (case review) then a collective judgement after a panel discussion to reach a consensus^11^^,^^18^^,^^19^^,^^27^^,^^34^^,^^47^^,^^51^^,^^55^^,^^61^^,^^70^^,^^74^ versus review meetings only^15^^,^^24^; cause of death recorded using well-known classifications and/or selected contributory factors from a framework^14^, ^15^, ^16^, ^17^, ^18^, ^19^^,^^21^^,^^24^^,^^25^^,^^38^^,^^39^^,^^43^^,^^45^^,^^47^^,^^53^^,^^54^^,^^56^^,^^58^, ^59^, ^60^^,^^62^^,^^65^^,^^68^; grading of suboptimal care using an established grading system such as the CESDI^3^^,^^10^^,^^18^^,^^19^^,^^22^^,^^24^^,^^30^^,^^35^^,^^36^^,^^47^^,^^49^^,^^51^^,^^53^^,^^60^^,^^61^^,^^65^^,^^66^^,^^69^^,^^70^^,^^74^^,^^75^; external reviews being conducted for quality assurance^15^^,^^18^^,^^27^^,^^46^^,^^61^; creating action plans and follow up plans^3^^,^^15^, ^16^, ^17^^,^^24^^,^^34^^,^^37^^,^^40^^,^^45^^,^^48^^,^^50^^,^^61^^,^^63^^,^^67^^,^^73^^,^^75^; assessing if the death was avoidable, if there was suboptimal care, usually using a grading system or a criteria^3^^,^^12^^,^^13^^,^^15^^,^^17^^,^^20^, ^21^, ^22^^,^^25^, ^26^, ^27^^,^^29^, ^30^, ^31^, ^32^, ^33^^,^^35^^,^^36^^,^^38^^,^^39^^,^^42^^,^^43^^,^^45^^,^^47^^,^^53^^,^^55^^,^^58^, ^59^, ^60^^,^^62^^,^^65^^,^^67^^,^^70^^,^^72^^,^^74^^,^^75^; evaluation against criteria/guidelines^24^^,^^27^^,^^36^^,^^44^^,^^55^^,^^58^^,^^71^^,^^75^ versus evaluation against personal knowledge, experience, and culture^56^^,^^61^^,^^71^; having separate administrative staff or a chair to facilitate reviews^24^^,^^35^^,^^36^^,^^77^; lead presenter presents the allocated case^10^^,^^34^^,^^36^^,^^45^^,^^58^^,^^60^^,^^61^^,^^70^^,^^75^; findings distributed in multiple languages^61^; and grade/comment on the quality of clinical notes.^55^^,^^62^^,^^74^

All included studies produced outputs (data or information) that resulted from the stillbirth review. The themes identified were: risk, protective and modifiable risk factors for stillbirth (maternal, fetal and, obstetric); trends in stillbirth (e.g. changes in stillbirth rate); counts or proportion of causes of stillbirth (cause of death); details and grading of suboptimal care (what, when, who); proportion of stillbirths where post-mortem examinations were conducted; recommendation of actions to be implemented to reduce future stillbirths; and quality of case notes (record-keeping).

Not all reviews led to an outcome. Identified in 14 studies, the following short-term and medium-term outcomes were observed: providing cost-effective ways of increasing mothers’ awareness of stillbirth risk factors and level of knowledge about recognising the need to seek care^50^; improving referral pathways and record keeping to streamline the quality management process of providing optimal care^3^^,^^25^^,^^50^^,^^53^^,^^59^^,^^63^^,^^67^; increasing and improving clinical tests available and medical capacity (equipment, available health care professionals within the department) within the facilities^3^^,^^25^^,^^30^^,^^38^^,^^50^^,^^51^^,^^54^^,^^59^^,^^62^^,^^65^^,^^67^^,^^75^; and providing training to health care professionals to increase their knowledge and medical skills.^25^^,^^38^^,^^50^^,^^54^^,^^59^^,^^67^ None of the studies reported any impact on the incidence of stillbirth which is difficult to attribute to the review process alone.

Almost all included studies (97%) had clear criteria for inclusion in the review except a study from India by Bhatt^64^ and an Indonesian study by Supratikto et al.*,*^66^ which only explained the inclusion criteria of maternal deaths occurring in a facility setting. In order to measure stillbirth in a standard and reliable way for all cases included, the review should use a consistent definition of stillbirth applicable to all cases or consistent experts' assessment methods. This was seen in 71% of included studies, but could not be determined for 25% of the included studies. Three studies did not measure stillbirth in a standard and reliable way for all cases.^16^^,^^43^^,^^49^ Most of the studies (85%) were able to demonstrate which source of data they used for identification of the stillbirth and 82% of the studies had consecutive inclusion of stillbirths to reduce selection bias. At a minimum, the stillbirth review should collect some important demographic characteristics of the mothers who had a stillbirth to compare against well-known risk factors for stillbirth. However, 46% of the included studies did not include information such as age of the mother, parity, gestational age at birth, pregnancy complications and number of antenatal visits during pregnancy. While all included studies in this systematic review stated that their stillbirth review process evaluated the quality of care and/or avoidable factors, 35% of the included studies did not discuss the details of suboptimal care. In addition, these studies did not explain when the risk factors could have been avoided in relation to the pregnancy period (e.g. antenatal, labour and delivery) and who could have addressed these factors (e.g. obstetric staff, social care staff, mothers). It is important to consider what can be improved in the facility setting to prevent any future stillbirth, and this is one of the main aims of conducting a stillbirth review. While all studies may have identified the weakness in their facilities and made recommendations, most of the studies (79%) did not describe whether they actioned any recommendations as a result of the review outcomes. Lastly, 90% of the studies reported the presenting sites' or clinics’ information and 96% used appropriate statistical analysis methods to analyse their data. Quality assessment of the included studies are visualised in Supplementary material: Appendix 6.

Facilitators and barriers in order to successfully implement a stillbirth review were identified from 14 included studies.^3^^,^^25^^,^^30^^,^^38^^,^^50^^,^^51^^,^^53^^,^^54^^,^^59^^,^^62^^,^^63^^,^^65^^,^^67^^,^^75^ These are described in Table 4. Facilitators and barriers identified from the studies were categorised into three main themes: resources, expertise, and commitment. These three themes are connected in the sense that, the facilities with greater commitment (e.g. leadership buy-in) were more willing to provide resources (e.g. funds, training, administrative staff) to implement and strengthen their stillbirth review process. This further encouraged staff to be more committed to the ethos and aims of the review process. With adequate training provided (resources), staff could gain confidence in communication skills which could enable them to build trust with mothers, as well as focus on a blame-free culture when conducting the reviews. Adequate resources will help to engage experts from different specialities to create a multidisciplinary panel. In addition, with greater commitment from the staff and available tools and guidelines given for the purpose of the stillbirth review, better quality data will be collected, and adequate quality data will be available to conduct a thorough review. Better quality of data collected is important to address barriers such as poor or missing data. Another important barrier identified is poor or missing post-mortem data. Staff training could improve data quality, but engagement with parents will be required to receive approval for a post-mortem. Engaging with parents in the review process will also enable staff to collect more robust demographic data and create opportunity for parents to provide additional relevant information pertinent to the review and find out why their baby died after the review process is concluded.

**Table 4:** Facilitators and barriers in implementing a successful stillbirth review.

Themes	Facilitators (frequency of studies reporting)	Barriers (frequency of studies reporting)
**Resources** • Facilitators (n = 22) • Barriers (n = 21)	- Administrative staff and supporting researchers dedicated to the stillbirth review (n = 3) - Training in conducting stillbirth review (n = 3) - Availability of neutral chairs in the meetings to reduce the risk of bias (consistent review methods) and create a safe environment for panels to discuss cases (n = 2) - Data collection and stillbirth review tools or frameworks (n = 6) - Adequate data available (n = 2) - Funding (n = 6)	- Poor or missing post-mortem examination data (n = 3) - Amount of time spent in gathering data from multiple sources and inconsistencies in data between multiple sources (n = 5) - Inadequate capacity and resources (Human Resources, no tools/frameworks to collect data or conduct reviews, or summaries the results of the reviews) (n = 7) - Changes in guidelines since the stillbirth occurred (n = 1) - Lack of training in collecting data and/or conducting reviews (n = 2) - Language barriers between the mothers/families and the health care professionals to collect necessary data (n = 1) - Limited funds for conducting reviews and implementing the action points from the review results (n = 2)
**Expertise** • Facilitators (n = 13) • Barriers (n = 11)	- Multidisciplinary review teams/panels (MDT) (n = 6) - External reviewers as part of the MDT (n = 2) - Supervision from the senior members of the review team (n = 3) - Trust between hospital staff and mothers/families to collect data required (n = 2)	- Inconsistencies of grading/identification of suboptimal care between reviewers (n = 2) - Poor record keeping to document events adequately (n = 7) - Emotional involvement of the panel in the cases (n = 1) - Lack of confidentiality leading to pressure on certain departments/types of health care professional (n = 1)
**Commitment** • Facilitators (n = 16) • Barriers (n = 7)	- Equal opportunities to participate regardless of the level of seniority or job titles in the panel (n = 2) - Anonymous, blame-free, no-fault review environment (n = 6) - Strong commitment of the public care/research sectors and participation of the private health care sectors by sharing common aims of reducing stillbirth and improving care (n = 5) - Number of stillbirths (smaller the size, easier to review all cases) (n = 1) - Leadership buy-in (n = 2)	- Perceived differences in status between representatives of different health professionals in the review panel (e.g. midwives vs. senior obstetricians) (n = 1) - Lack of leadership buy-in (n = 2) - Lack of motivation of the health care professionals to reflect their care (n = 2) - The volume of deaths when this is large in number (n = 2)

**Fig. 3:**
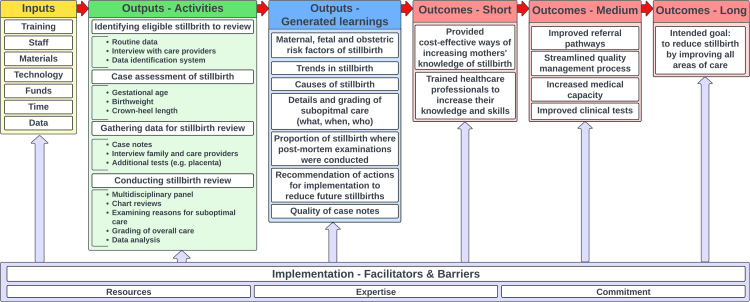
Logic model of stillbirth reviews.

A logic model was developed after carrying out the narrative synthesis. Fig. 3 maps out how a facility-based stillbirth review can work effectively with inputs and activities (primary output), which result in generated learnings (secondary outputs) and outcomes. However, the relationship between inputs, outputs, outcomes and impact needs to be understood in the context of the facilitators and barriers to implementing a successful stillbirth review. For example, while the “proportion of stillbirth where post-mortem examinations were conducted” is not an output or learning to reduce stillbirth, this was included in the logic model because having poor or missing post-mortem examination data was identified as a barrier in conducting stillbirth reviews in some studies^17^^,^^69^ (see Table 4). Autopsy and placental histology are important examinations to help identify the cause of stillbirth.^79^, ^80^, ^81^

The generated learnings (secondary outputs) have the capacity to lead to short-term outcomes such as new training programme for staff, and medium-term outcomes such as improved referral pathway to improve the quality of care in the facilities. However, long-term outcomes or impact on reducing the incidence of stillbirth will take longer to materialise, and it will be difficult to attribute them solely or directly to the review process alone.

## Discussion

This systematic review identified different types and methods of facility-based stillbirth review processes around the world and a logic model was built from the extracted evidence to describe what a successful stillbirth review process could look like. The search identified 68 studies that described the methods used to conduct a facility-based stillbirth review from 39 countries (17 HICs and 22 LMICs). There were reviews conducted at various levels (district, state and national), and three types of stillbirth review were identified (audit, review and confidential enquiry) although most reviews did not include the salient features for the review process used, which led to a mismatch between the author description of the type of reviews and as the actual salient features of the process. Reviews from many countries had agreed that the aim of the stillbirth reviews were to prevent stillbirth, and to improve all aspects of care by making adequate and informed recommendation guided by clear local/national/international criteria or practices.

In terms of data sources for identifying stillbirths for the review, routine data was the most popular method. Some studies additionally conducted interviews with care providers, and many studies implemented a specific data identification system to build a stillbirth database only for the purposes of the review. Case assessment using stillbirth definition was used in 48 out of 68 studies. However, there was variation among the studies in the inclusion of these characteristics, and in the ranges included, regardless of the type of country involved. All stillbirth review processes identified in this study generated outputs that can lead to outcomes. Short-term and medium-term outcomes were reported in 14 studies, but the impact of the review process on reducing stillbirth, which is more difficult to establish, was not reported in any study. Facilitators and barriers identified from 14 studies focused on three main themes: resources, expertise, and commitment. The generated learnings from the stillbirth review processes inform the action plans and allow facilities to consider where the changes should happen to improve the quality of care in the facilities, enabling positive short-term and medium-term outcomes.

Stillbirth review process is not standardised in many countries at a local/national level, despite the known value of such review programmes in reducing stillbirth rates over time and identifying gaps in care.^82^ Although it was important to have a standard process across all regions in a country, this might nevertheless be only possible in a small country like the UK with not much variation in the healthcare system and healthcare provision across the regions. Standardisation might not be possible in large countries with variable systems such as the United States, India, and others, which do not have a standard care provision system for all in health facilities. This was evident in a national programme, the Fetal Infant Mortality Review Program from the United States, as guidelines existed at the national level, yet different States were implementing the stillbirth review using different methods that uniquely work for each State.^44^^,^^46^ This is in contrast to the programme that exists in the UK where the confidential enquiries and review methods employ standardised tools that can be used in any region in order to facilitate standardised national monitoring.^25^^,^^36^ It was observed, however, that even with a national programme in place, without a robust environment to implement the programme, the uptake of stillbirth review can be very low in areas that do not have adequate resources and support. For example, when perinatal death was added as a component to the National Maternal Death, Surveillance and Response in Kenya in 2016, Bungoma was the only county that reviewed more than 50% of the deaths. A study found that the technical and training support from UKAID was the main reason why the programme could be successfully implemented and strengthened in this county compared to others that did not receive this type of support.^67^

There was a lack of application of core principles of an audit process within studies that defined their review processes as “audits”, especially as many studies^13^, ^14^, ^15^, ^16^^,^^18^^,^^20^^,^^21^^,^^23^^,^^26^, ^27^, ^28^, ^29^^,^^31^, ^32^, ^33^, ^34^^,^^45^^,^^52^^,^^57^^,^^60^^,^^61^^,^^64^^,^^68^^,^^71^^,^^72^^,^^77^ lacked implementation of changes based on outputs and failed to ensure a continuous audit cycle of re-evaluation. Reporting on how changes are made by making adjustments in clinical practices based on stillbirth review outputs can be challenging, as it can be a gradual and time-consuming process that requires both service-level and policy-level support and many other processes and findings may be involved beyond reviews. However, there is a need for clear guidelines on how to measure the impact of such changes. The continuous audit cycle of re-evaluation can be also challenging due to the cost (time and financial) associated with conducting a stillbirth review; particularly compromises to clinical care could be counter-productive if health care professionals are required to spend clinical time participating in the review meetings without providing additional resources to pay for this time.^5^

For the studies that defined their stillbirth review process as “reviews”, dissemination of key findings and learning points to relevant staff were not observed in many. The dissemination of information should activate knowledge translation within the hospitals/units which then forms part of the knowledge-to-action cycle, and can support promoting sustainable changes that improve clinical practices.^83^ Therefore, it is crucial for studies to enable effective dissemination of learning points in the future and promoting them through training platforms that are already available in their facilities.^84^ While the United States Department of Veterans Affairs Root Cause Analysis Tool states that training and dissemination are a weak means of changing either behaviour or outcomes, many studies from LMICs found that these types of actions are an effective ways to change clinical practices to reduce suboptimal care in health facilities.^85^ This may be due to differences in health systems and/or provider knowledge and practices in HICs versus LMICs, as LMICs may not usually have the facilities or practices in place to conduct training for the healthcare professionals or disseminate significant information about why a baby died and therefore when this happens it may have more impact. The results of this systematic review also revealed that a stillbirth review process could be an amalgamation of different types rather being confined to the salient features that are thought to be desirable for a specific type, and different processes will most definitively comprise a variable mix of audit, review and confidential enquiry components. It is important, however, to recognise that implementing a stillbirth review in a facility-based setting is a ‘test and learn’ process and if the desired components of stillbirth review are missing, it does not mean that the review methods are ineffective. As seen in early maternal mortality reviews in Ireland, UK and the United States, from 1920s to 1940s, the processes initially did not examine the standard of care, however, this was later revised and more structured approaches to maternal mortality reviews were established.^9^ Understanding that stillbirth review is relatively new compared to maternal mortality reviews, stillbirth review processes are likely to adapt and improve over time. Different countries/districts/hospitals will have different stillbirth review processes to optimise their resources and contextualise their healthcare system, cultural and social norms.

The definition used to assess stillbirth can vary greatly between studies, and there is a need to develop and adopt a universal definition of stillbirth to facilitate meaningful comparison of stillbirth rates between regions and to correctly identify risk, protective, and modifiable risk factors for stillbirth, even in HICs.^86^ This has been also discussed extensively in a previous systematic review by Aminu et al., and in the latest 2023 report by the United Nations Inter-agency Group for Child Mortality Estimation.^87^^,^^88^ The minimum gestational age, birthweight and crown-heel length ranges used to define stillbirth were lower in HICs than the range in LMICs. This could be because LMICs prefer to use the WHO definition of stillbirth for international comparison and reporting.^89^ The rationale for moderating the definition of stillbirth cut-off for international comparison to 1000 g or more at birth, or ≥ 28 completed weeks of gestation, or attainment of at least 35 cm crown-heel length could be to ensure comparability as many LMICs may not capture late fetal deaths accurately and data is limited.^90^^,^^91^ In addition, in countries where neonatal intensive care units are limited, babies below the gestational age of 28 weeks may not be viable when delivered prematurely, unlike HICs, where facilities are available, and lower gestational age babies can survive with adequate neonatal care and treatment.^92^

There were unique components of stillbirth review in some studies that showed positive effects on achieving their aims which can be utilised in future designs of stillbirth review. Some studies explicitly stated how reviewers were trained in the review process before they started participating in a review panel.^18^^,^^50^ Effective training will not only reduce measurement errors from between-reviewer variation, but also allows reviewers to prioritise the guiding principles for stillbirth reviews, for example, focusing on improvements to health systems and not individuals (no blame policy). Sharing review-related data a few weeks prior to the panel review is another effective way to promote the review meetings and to stay focused on the agenda without panels feeling unprepared or rushed because they have not had enough time to absorb the amount of information available in the case notes. Allowing review panels to study the cases before the meetings allows them to understand the cases fully and helps them in gaining confidence to participate in the discussion sessions. This may also create a system where there is a lead presenter to present an allocated case in each session to open up the discussion more naturally during the review meeting.

Some facilities may have structured forms or checklists that guide the stillbirth review process, while some only conduct the review via discussion with the panel. Nonetheless, having well-known classifications to assess the cause of death or a framework to select contributory factors, could increase the consistency between the reviewers and the extent to which consensus is reached. Aminu et al., however, notes that there are over 35 classification systems for stillbirth and none of them are adopted globally.^87^ In addition, some argue that even the widely known classification systems such as Extended Wigglesworth and Amended Aberdeen are considered limited in classifying stillbirth.^87^^,^^93^ Since creating action plans and follow-up plans are important steps to implement changes based on stillbirth review outputs, having a clear reporting of suboptimal care (what, when, who), grading suboptimal care using an established grading system (e.g. CESDI^38^) and assessing if it contributed to death could equip review panels to select and justify appropriate action plans successfully.

As identified in the facilitators to implementation of the identified stillbirth reviews, having separate administrative staff or a chair to facilitate reviews could be effective in reducing the workload and increasing motivation and consistency between review meetings. Furthermore, as data quality was identified as one of the barriers to implementation, an opportunity for review panels to grade or comment on the quality of clinical notes can increase awareness among the health care professionals of the importance of accurate and detailed record keeping of cases.

The strength of this systematic review is that this is one of the first, of which we are aware, to critically appraise and synthesise the methods used for facility-based stillbirth review process across the world, in detail. We used a robust methodology by including independent reviewers for screening, extracting and quality assessment. Since there are high levels of heterogeneity between included studies due to the nature of the aim of this systematic review, such as methodological characteristics, as well as clinical heterogeneity such as diversity in the populations (different regions and countries), several tools for risk of bias assessment and different types of frameworks for narrative synthesis were considered and piloted before finalising the most applicable methods to appraise the included studies.

While using a logic model for narrative synthesis was deemed most appropriate for this study, logic models represent a linear model of how processes should work. Since the sequence of implementing a stillbirth review in the real world is not linear, and assumptions are often not met, the logic model proposed in this study should be interpreted with flexibility when designing a stillbirth review process. Moreover, the proposed logic model in this study assumes that this model would apply to all settings within and across countries. Whereas the same audit process in differing contexts may also involve different outcomes, reflecting differing reasons for stillbirth rates being elevated. Therefore, users should critically appraise the logic model and update their methods, if necessary, by considering the contextual settings and understanding the complexity in implementing a stillbirth review in their setting.

In addition, studies reporting on the stillbirth review process may not always reach mainstream sources of literature, and despite efforts made to retrieve studies by searching published and unpublished or grey literature, the systematic review might not have identified every possible method used for facility-based stillbirth review process across the world.

Finally, this study compared different types of reviews using Helps et al.’s interpretation of the desired components of audits, reviews, and confidential enquiries as standard guidance.^9^ However, Helps et al. focused on the types and evolution of audits and reviews in HICs alone when deriving the desired components. Considering this study looks at stillbirth review processes in both HICs and LMICs, there are limitations on using Helps et al.’s interpretation of salient features for different types of review processes because their method may not fully reflect LMICs' health systems, which may differ from HIC's systems for standard care provision.

In conclusion, this systematic review identified different types and methods of facility-based stillbirth review processes implemented around the world and a logic model was built from the extracted evidence to describe what a successful stillbirth review process might encompass. The findings this comprehensive review were used to develop a logic model which could be used as a guide or contextually adapted by health facilities to improve their stillbirth review process. This systematic review raises important questions about what the gold standard stillbirth review process is and how this can be achieved, and whether we can reach a global consensus. The logic model developed from this review is the first step in trying to achieve a global standard for stillbirth review. It does however need to be used to assess its utility and usability and reach consensus about its use as a global standard for stillbirth review. Moreover, since most stillbirths (98%) occur in LMICs, there is an urgent need to introduce standardised stillbirth review processes in more LMICs, while considering the facilitators and barriers, resources required, training and support needs.

## Contributors

YYB, NR, JJK, ML and MN were responsible for conceptualisation and methodology. YYB was responsible for data curation, funding acquisition, visualisation, and writing the first draft of the paper. YYB, UGA and DBT were responsible for formal analysis, and investigation. NR was responsible for library resources. YYB and MN were responsible for project administration. YYB, NR and MN were responsible for validation. NR, JJK, ML and MN were responsible for supervision. All authors were responsible for reviewing and editing the drafted paper. YYB, UGA, DBT have accessed and verified the underlying data and all authors were responsible for the decision to submit the manuscript.

## Data sharing statement

All data relevant related to the study are included in the article or uploaded as supplementary information.

## Declaration of interests

MN is a member of data safety and monitoring board for PREVENT study, a multicentre study on fetal brain injury in South Asia led by Prof Sudhin Thayyil, Imperial College, London (2021 onwards). All other authors declare no competing interests.

## Appendix A Supplementary data

Appendices 1–6 and References
