# Supplementary material for: Facility-based stillbirth review processes used in different countries across the world: a systematic review
Source: eClinicalMedicine. 2023 Apr 27;59:101976. doi: 10.1016/j.eclinm.2023.101976 (PMC10173150; doi:10.1016/j.eclinm.2023.101976)
Supplement: Appendices 1–6 and References [file mmc1.pdf]

Table of Contents

Appendix 1: Search terms used for each database 2
Appendix 2: Data extraction template 4
Appendix 3: Quality assessment template 9
Appendix 4: Components of stillbirth review identified in the methods 11
Appendix 5: Included studies and the extracted data 13
Appendix 6: Quality assessment of the included studies 26
References 29

Appendix 1: Search terms used for each database

**Medline (Ovid MEDLINE® Epub Ahead of Print, In-Process & Other Non-Indexed Citations, Ovid MEDLINE® Daily and Ovid MEDLINE®) 1946 to present**	
#	Searches
1	(fetal mortality/ or perinatal mortality/) and (clinical audit/ or medical audit/)
2	(Pregnancy Complications/mo or Stillbirth/) and (clinical audit/ or medical audit/)
3	((fetal or foetal or perinatal) adj3 (mortality or death?)).ti,ab. and (clinical audit/ or medical audit/)
4	stillbirth?.ti,ab. and (clinical audit/ or medical audit/)
5	(fetal mortality/ or perinatal mortality/) and (review* or audit* or meeting? or enquir* or inquir*).ti,ab.
6	(Pregnancy Complications/mo or Stillbirth/) and (review* or audit* or meeting? or enquir* or inquir*).ti,ab.
7	(((death? or mortality) adj3 (review* or audit* or meeting? or enquir* or inquir*)) and (fetal or foetal or perinatal)).ti,ab.
8	(stillbirth? adj5 (review* or audit* or meeting? or enquir* or inquir*)).ti,ab.
9	((confidential enquir* or confidential inquir*) and ((fetal or foetal or perinatal) adj3 (mortality or death?))).ti,ab.
10	((confidential enquir* or confidential inquir*) and stillbirth?).ti,ab.
11	(cemach or cmace or cemd or cmde).ti,ab.
12	(saving mothers lives or making pregnancy safer or making childbirth safer).ti,ab.
13	((near miss* or significant event* or critical event* or critical incident?) and (fetal or foetal or perinatal or stillbirth) and (review* or audit* or meeting? or enquir* or inquir*)).ti,ab.
14	1 or 2 or 3 or 4 or 5 or 6 or 7 or 8 or 9 or 10 or 11 or 12 or 13
15	exp Animals/ not humans/
16	14 not 15
17	(review or meta analysis or news or comment or editorial).pt. or cochrane database of systematic reviews.jn. or comment on.cm. or (systematic review or literature review).ti.
18	16 not 17

**Embase 1974 to present**	
#	Searches
1	(fetus mortality/ or perinatal mortality/) and medical audit/
2	((*Pregnancy Complications/ and (*"cause of death"/ or *mortality/)) or exp *fetus death/) and medical audit/
3	((fetal or foetal or perinatal) adj3 (mortality or death?)).ti,ab. and medical audit/
4	stillbirth?.ti,ab. and medical audit/
5	(fetal mortality/ or perinatal mortality/) and (review* or audit* or meeting? or enquir* or inquir*).ti,ab.
6	((*pregnancy complication/ and (*"cause of death"/ or *mortality/)) or exp *fetus death/) and (review* or audit* or meeting? or enquir* or inquir*).ti,ab.
7	(((death? or mortality) adj3 (review* or audit* or meeting? or enquir* or inquir*)) and (fetal or foetal or perinatal)).ti,ab.
8	(stillbirth? adj5 (review* or audit* or meeting? or enquir* or inquir*)).ti,ab.
9	((confidential enquir* or confidential inquir*) and ((fetal or foetal or perinatal) adj3 (mortality or death?))).ti,ab.
10	((confidential enquir* or confidential inquir*) and stillbirth?).ti,ab.
11	(cemach or cmace or cemd or cmde).ti,ab.
12	(saving mothers lives or making pregnancy safer or making childbirth safer).ti,ab.
13	((near miss* or significant event* or critical event* or critical incident?) and (fetal or foetal or perinatal or stillbirth) and (review* or audit* or meeting? or enquir* or inquir*)).ti,ab.
14	1 or 2 or 3 or 4 or 5 or 6 or 7 or 8 or 9 or 10 or 11 or 12 or 13
15	(exp animals/ or nonhuman/) not human/
16	14 not 15
17	(editorial or letter or note or "review").pt. or cochrane database of systematic reviews.jn. or (systematic review or literature review).ti.
18	16 not 17

**Global Health <1973 to 2021 Week 49>**	
#	Searches
1	(perinatal mortality/ or stillbirths/) and (review* or audit* or meeting? or enquir* or inquir*).ti,ab.
2	pregnancy complications/ and ("causes of death"/ or mortality/) and (review* or audit* or meeting? or enquir* or inquir*).ti,ab.
3	(((death? or mortality) adj3 (review* or audit* or meeting? or enquir* or inquir*)) and (fetal or foetal or perinatal)).ti,ab.
4	(stillbirth? adj5 (review* or audit* or meeting? or enquir* or inquir*)).ti,ab.
5	((confidential enquir* or confidential inquir*) and ((fetal or foetal or perinatal) adj3 (mortality or death?))).ti,ab.
6	((confidential enquir* or confidential inquir*) and stillbirth?).ti,ab.
7	(cemach or cmace or cemd or cmde).ti,ab.
8	(saving mothers lives or making pregnancy safer or making childbirth safer).ti,ab.
9	((near miss* or significant event* or critical event* or critical incident?) and (fetal or foetal or perinatal) and (review* or audit* or meeting? or enquir* or inquir*)).ti,ab.
10	1 or 2 or 3 or 4 or 5 or 6 or 7 or 8 or 9

**WHO Global Health Medicus (globalindexmedicus.net)**	
#	Searches
1	(fetal OR foetal OR perinatal) AND (death OR deaths OR mortality) AND (audit OR audits OR enquiry OR enquiries OR inquiry OR inquiries OR meeting OR meetings)
2	(death review OR mortality review OR death reviews OR mortality reviews) AND (fetal OR foetal OR perinatal)
3	(stillbirth OR stillbirths) AND (audit OR audits OR enquiry OR enquiries OR inquiry OR inquiries OR meeting OR meetings)

4	confidential enquiry OR confidential enquiries OR “confidential inquiry” OR “confidential inquiries” OR cemach OR cmace OR cemd OR cmde OR saving mothers lives OR making pregnancy safer OR making childbirth safer
5	(near miss OR significant events OR critical events OR critical incidents OR significant event OR critical event OR critical incident) AND (audit OR audits OR enquiry OR enquiries OR inquiry OR inquiries OR meeting OR meetings) AND (fetal OR foetal OR perinatal)
6	1 OR 2 OR 3 OR 4 OR 5

**CINAHL (EBSCOHost) [1982-present]**	
#	Searches
S12	S1 OR S2 OR S3 OR S4 OR S5 OR S6 OR S7 OR S8 OR S9 OR S10 OR S11
S11	TI (("near miss*" or "significant event*" or "critical event*" or "critical incident?") and (foetal or perinatal) and (review* or audit* or meeting? or enquir* or inquir*)) or AB (("near miss*" or "significant event*" or "critical event*" or "critical incident?") and (foetal or perinatal) and (review* or audit* or meeting? or enquir* or inquir*))
S10	TX saving mothers lives or making pregnancy safer or making childbirth safer
S9	TX cemach or cmace or cemd or cmde
S8	TX ((confidential enquir* or confidential inquir*) and (stillbirth*))
S7	TX ((confidential enquir* or confidential inquir*) and ((foetal or perinatal) N3 (mortality or death*)))
S6	TX ((stillbirth*) N5 (review* or audit* or meeting* or enquir* or inquir*))
S5	TI (((death* or mortality) N3 (review* or audit* or meeting* or enquir* or inquir*)) and (foetal or perinatal)) or AB (((death* or mortality) N3 (review* or audit* or meeting* or enquir* or inquir*)) and (foetal or perinatal))
S4	(((MH "Pregnancy Complications+/MO") OR (MH "Perinatal Death"))) AND (TI ((review* or audit* or meeting? or enquir* or inquir*)) or AB ((review* or audit* or meeting? or enquir* or inquir*)))
S3	((stillbirth*)) AND (MH "Audit")
S2	(((foetal or perinatal) N3 (mortality or death*))) AND (MH "Audit")
S1	((MH "Pregnancy Complications+/MO") OR (MH "Perinatal Death")) AND (MH "Audit")

**WHO database (who.int)**
stillbirth site:who.int.
stillbirth review site:who.int.
stillbirth audit site:who.int
perinatal site:who.int.
perinatal death review site:who.int.
perinatal death audit site:who.int.
confidential inquiries site:who.int.
foetal death review site:who.int.
foetal death audit site:who.int.

**Google Scholar (scholar.google.com)**
(stillbirth OR perinatal mortality OR perinatal death) (audit OR chart review OR mortality review OR death review OR confidential enquiry)

**ProQuest Dissertations & Theses Global (proquest.com)**
ab(Stillbirth OR perinatal mortality OR perinatal death) AND ab(audit OR chart review OR mortality review OR death review OR confidential enquiry)

Appendix 2: Data extraction template

General information
1. First author and Year
E.g. Boo, 2021

2. Country in which the study conducted
Please **HIGHLIGHT** the country where the stillbirth review was/has been conducted. Some papers describe several countries, e.g. EuroNatal Working Group, which were Belgium, Denmark, the UK, Finland, Greece, Netherlands, Norway, Spain and Sweden. **Please select all that apply.**

1. Australia
2. Bangladesh
3. Brazil
4. Belgium
5. Denmark
6. Ethiopia
7. Fiji
8. France
9. Finland
10. Ghana
11. Guadeloupe
12. Greece
13. India
14. Indonesia
15. Israel
16. Italy
17. Jordan
18. Kenya
19. Malawi
20. Moldova
21. Mozambique
22. Netherlands
23. New Zealand
24. Norway
25. Papua New Guinea
26. Rwanda
27. Sierra Leone
28. Singapore
29. South Africa
30. Spain
31. Sudan
32. Sweden
33. Tanzania
34. Thailand
35. Timor-Leste
36. Uganda
37. United Kingdom
38. United States
39. Zambia
40. Zimbabwe
41. Other: specify

3. Notes
If you have any additional notes you want to write down.

The facility-based stillbirth review process

4. Aim

E.g. to present the analysis and assess avoidable factors associated with stillbirths identified in an ongoing trial.
E.g. In 2016, perinatal death was added as a component to the national MDSR guidelines in Kenya
E.g. to improve the quality of care provided by a maternity service in rural Africa and to use the results of the review to inform the design of interventions (structural and functional rearrangement of the maternity service district-wide, writing and implementing protocols of care for local use, and regular in-service education).

5. Funding

E.g. Financed by the Regional Health Agency of Ile-de-France.
E.g. Conducted under the UKAID funded Maternal and Newborn Health Initiative (MANI) project in Kenya. The MANI project and staff members working and funded by UKAID under this project undertook te review as part of documenting learnings.

6. Level

Please **<u>HIGHLIGHT</u>** your selection (**select only one**)

1. Local/hospital(s)
2. District/state
3. National
4. International
5. Other

7. Details of the hospital/regions

Name of the hospitals, number of hospitals involved, number of states involved that can be identified. Sometimes, due to the confidentiality and sensitive nature of reporting of stillbirth, the paper may have kept this information vague.

E.g. district hospital and a tertiary referral hospital in Kigali, Rwanda
E.g. midwife-managed unit within the Aberdeen Trial
E.g. 10 peri-urban and rural health centers in East New Britain and Madang provinces

8. Type

Please **<u>HIGHLIGHT</u>** your selection (**select only one**) according to how the study described themselves.

1. Audit
2. Review
3. Confidential enquiries

9. What did the process (audit/review/confidential enquiries) entail?

To guide "Examine reasons for cases for suboptimal care and/or avoidable factors", the review should look into components of care that were judged to be substandard (e.g. detection, resources, organisational or communication). If the review inquired if the suboptimal care occurred or not, but there were no details about what it entailed, then this review does not "Examine reasons for cases for suboptimal care and/or avoidable factor".

Please **<u>HIGHLIGHT</u>** your selection (**select all that apply**)

1. Data collection
2. Case assessment
3. Implementation of changes based on outputs
4. Re-evaluation (i.e. a continuous cycle)

5. A collaboration between different specialities in obstetrics, midwifery, and pathology (i.e. the multidisciplinary team)
6. Examine reasons for cases for suboptimal care and/or avoidable factors
7. Dissemination of key findings and learning points to all relevant clinical staff
8. Anonymous review
9. Other

10. Duration/Time
E.g. 2020-2021, Jan-July 2021

11. Source of data
Examples, usually applicable if the review was done in a local facility setting and review was conducted prospectively:

E.g. Hospital records and interviews

Examples, usually applicable if the review was done in a confidential inquiry setting or retrospective review as part of a research project:

E.g. the national Swedish Medical Birth Register
E.g. all stillbirths identified in Severe adverse events reports from the Women and Newborn Trial of Antenatal Interventions and Management (WANTAIM) trial

12. Identification of stillbirth
E.g. A stillbirth was defined as a fetal death after at least 22 completed weeks of gestation or weighing 500 g or more at birth.

How the stillbirth was defined. Since stillbirth is quite straightforward to diagnose, some papers might have not listed how they identified stillbirth, and may just state the source of data. If this is the case, please put N/A.

13. Data collection method
E.g. using a questionnaire containing both closed and open-ended questions. The first part of the questionnaire included information on the socioeconomic characteristics of the mothers and their households. The second part included data on routine antenatal screening and care, pregnancy complications and their management, characteristics of labour and delivery, condition of the baby at birth (alive or stillbirth) and afterwards, Apgar score, management of the newborn’s illness that led to a fatal outcome, and time and causes of death. The third part of the questionnaire comprised information obtained from interviews with mothers and healthcare providers.

E.g. Narratives based on individual records were written for each birth. They contained in-depth descriptions of relevant time-related events, including such elements as medical history, social conditions, communication, surveillance, interventions and outcome.

14. Who collected the data
E.g. one nurse, one midwife, and one doctor
E.g. one member of the research team

15. Who conducted the review
E.g. An expert panel of three obstetricians and one neonatologist
E.g. Audit committee were recruited from the
hospitals and included two obstetricians, two paediatricians,
and the main investigator, who is also a paediatrician

16. Definition of suboptimal care and/or avoidable factors
Not all studies have defined the suboptimal and/or avoidable factors. If this is the case, put N/A.

E.g. An error (e.g. incorrect labour ward management decision) or omission (e.g. failure to test for syphilis at antenatal booking) on the part of a member of the health service team (administrative staff, drivers, and laboratory technicians, as well as doctors and midwives)

17. Review tool or/and review method
Not all papers had a specific review tool or meeting. Some papers might have conducted a review by simply selecting a code in a database which then was reviewed by second or panel reviewers.

E.g. The abstracted chart data were reviewed independently by a panel.
E.g. Firstly, a medical investigator reviewed each death with the team that cared for the pergnant woman. Then, each case was reviewed, after removal of identifying information, by at least one peer assessor from an external panel.
E.g. Once one member of the research team conducted the initial analysis and coding of each case, a second reviewer reviewed and conducted an independent analysis and coding. The differences were resolved through discussion and consensus; adjudication where necessary were undertaken by a third reviewer. To further validate assigned codes, 10% of the cases were randomly selected, assessed, and coded independently by two obstetricians.

Outputs and outcomes
18. Output
Outputs are the data/information that results from the stillbirth review.

E.g. Perinatal Mortality Rate (PNMR), Proportion of death classified as avoidable.
E.g. Proportion and details of demographics/clinical information (e.g. gestational age, maternal condition) of mothers who had a stillbirth, cause of stillbirth, avoidable factors identified.

19. Outcome
Not all stillbirth reviews led to an outcome. Please state N/A if this is the case. Outcomes are different to outputs as outcomes are the short and medium benefits that a stillbirth review brings.

E.g. The review enabled the design and implementation of specific interventions targeted at areas of care causing unnecessary death.

Facilitators and barriers
20. Facilitators
Not all studies have identified facilitators. In this case, please put N/A.

E.g. awareness and training to mobilise health staff to attach prominence to and conduct reviews.
E.g. ensuring the focus remains on learning and improving quality of care rather than on placing blame or engaging in punitive measures

21. Barriers
Not all studies have identified barriers. In this case, please put N/A.

E.g. poor documentation and missing records led to difficulties in extracting data needed for the review.
E.g. Having the appropriate death review reporting templates in place at the facility level which align with national online forms.
E.g. the volume of deaths.

Appendix 3: Quality assessment template

1. Were there clear criteria for inclusion in the review?
The authors should provide clear inclusion (and exclusion criteria where appropriate) for the study participants. The inclusion/exclusion criteria should be specified (e.g., risk, stage of disease progression) with sufficient detail and all the necessary information critical to the study.

- Yes
- No
- Unclear
- Not applicable

2. Was the condition (stillbirth) measured in a standard, reliable way for all fetus included in the review?
The study should clearly describe the method of measurement of the condition. This should be done in a standard (i.e. same way for all patients) and reliable (i.e. repeatable and reproducible results) way.

- Yes
- No
- Unclear
- Not applicable

3. Were valid methods used for identification of the condition (stillbirth) for all fetus included in the review?
Sometimes stillbirths are not easily diagnosed or defined. If the outcomes (stillbirth) were assessed based on existing definitions or diagnostic criteria, then the answer to this question is likely to be yes. If the outcomes were assessed using observer reported, or self-reported scales, the risk of over- or under-reporting is increased, and objectivity is compromised. Importantly, determine if the measurement tools used were validated instruments as this has a significant impact on outcome assessment validity.

- Yes
- No
- Unclear
- Not applicable

4. Did the review have consecutive inclusion of stillbirth cases?
The completeness of a review contributes to its reliability. Studies that indicate a complete inclusion are more reliable than those that do not. As stated above, a review that states 'we included all cases with stillbirth who presented to our clinic between March 2005 and June 2006' is more reliable than a study that simply states 'we report a review of 24 stillbirths'.

- Yes
- No
- Unclear
- Not applicable

5. Was there clear reporting of the demographics of the mothers who had a stillbirth in the review?
Minimum, the stillbirth review should collect the following demographic information of the mothers. Please select **YES** if there are some or all of the information below reported in the report.

- Age (years)
- Parity (numbers)
- Gestational age at birth
- Pregnancy complications
- Antenatal visits
- live in Rural/urban area
- Any Socio-economic Status (SES) information

  - Yes
  - No
  - Unclear
  - Not applicable

6. Was there clear reporting of "quality of clinical care" of the stillbirth cases?

All included studies in this systematic review stated that their stillbirth review process assesses the quality of care and/or avoidable factors. Therefore, this question is asking if the review method lists the "quality of clinical care" in detail or not. So, if the quality of clinical care was listed with what kind of sub-optimal care during the pregnancy/labour occurred (details), then the answer to this question is likely to be yes. If it simply says 40% were found to have had components of suboptimal care but not details on what these were (e.g. no checkup for fetal movement).

- Yes
- No
- Unclear
- Not applicable

7. Were the outcomes or follow-up results of having a review process clearly reported?

This question is asking if having a review process allowed the facility to action any recommendations made as a result of the review outcomes (e.g. started providing training about the importance of fetal monitoring for nurses... etc. as this was found to be one of the suboptimal care factors that may contribute to stillbirth).

- Yes
- No
- Unclear
- Not applicable

8. Was there clear reporting of the presenting site(s)/clinic(s) demographic information?

Certain diseases or conditions vary in prevalence across different geographic regions and populations (e.g. sociodemographic variables between countries). However, stillbirth is a sensitive topic and the study might not report in detail where the review was conducted. If the paper has some/any information (e.g. was conducted in 3 hospitals) of the sites, please select Yes.

- Yes
- No
- Unclear
- Not applicable

9. Was statistical analysis appropriate?

If there was any statistical analysis, were the method listed in detail and were appropriate? The methods section of studies should be detailed enough for reviewers to identify which analytical techniques were used and whether these were suitable.

- Yes
- No
- Unclear
- Not applicable

Appendix 4: Components of stillbirth review identified in the methods

First author and year	A 1	A 2	A 3	A 4	R 1	R 2	R 3	C
**Reported type: Audit**								
Alderliesten, 2008[1]	✓	✓	X	X	✓	✓	✓	✓
Alyahya, 2021[2]	✓	✓	✓	✓	✓	✓	✓	X
Aminu, 2017[3]	✓	✓	✓	X	✓	✓	X	✓
Berge, 1991[4]	✓	✓	X	X	X	X	X	X
Bhatt, 1989[5]	✓	X	X	X	X	✓	✓	X
Bjellmo, 2019[6]	✓	✓	X	X	X	X	X	X
Copenhagen: WHO Regional Office for Europe, 2020[7]	✓	✓	X	X	✓	✓	X	X
de Caunes, 1990[8]	✓	✓	X	X	X	X	✓	X
Demise, 2015[9]	✓	✓	✓	✓	✓	✓	✓	X
Eksmyr, 1986[10]	✓	X	X	X	✓	✓	X	X
El Amin, 2002[11]	✓	✓	X	X	✓	✓	X	X
Eskes, 1993[12]	X	✓	X	X	✓	✓	X	X
Eskes, 2014[13]	✓	✓	✓	✓	✓	✓	✓	X
Essén, 2002[14]	✓	✓	X	X	X	✓	X	X
Flenady, 2021[15]	✓	✓	✓	✓	✓	✓	✓	X
Fossen, 1999[16]	✓	X	X	X	X	✓	✓	X
Furst, 1989[17]	✓	✓	X	X	X	✓	X	X
Govender, 2017[18]	✓	X	✓	X	X	✓	X	X
Han, 2018[19]	✓	✓	X	X	X	✓	X	✓
Kasengele, 2017[20]	✓	✓	✓	✓	X	X	✓	X
Kirabira, 2020[21]	✓	✓	✓	✓	✓	✓	✓	X
Maaløe, 2016[22]	✓	✓	X	X	X	X	X	X
Mbaruku, 2009[23]	✓	✓	X	X	✓	✓	X	X
Mdoe, 2022[24]	✓	✓	✓	✓	✓	✓	✓	X
Miranda, 1996[25]	✓	X	X	X	✓	✓	X	X
Moawad, 1990[26]	✓	✓	X	X	✓	✓	X	X
Musafili, 2017[27]	✓	✓	X	X	✓	✓	X	X
Omwodo, 2020[28]	✓	✓	X	X	✓	X	X	X
Pattinson, 1995[29]	X	✓	✓	X	X	✓	X	X
Raman, 2015[30]	✓	X	X	✓	X	✓	X	X
Sauvegrain, 2020[31]	✓	✓	X	X	X	✓	X	X
Sterpu, 2020[32]	✓	✓	X	X	✓	✓	X	X
Supratikto, 2002[33]	✓	✓	✓	✓	✓	✓	X	X
Vallely, 2020[34]	✓	X	X	X	X	✓	X	X
van Diem, 2010[35]	✓	✓	X	X	✓	X	X	X
van Diem, 2012[36]	✓	X	✓	X	✓	✓	✓	X
Ward, 1995[37]	✓	✓	X	✓	X	✓	X	X
Westergaard, 1997[38]	✓	✓	X	X	X	✓	X	✓
Wilkinson, 1997[39]	✓	✓	X	X	X	X	X	X
Wolleswinkel-van den Bosch, 2002[40]	✓	✓	X	X	✓	✓	X	X
D'Aloja, 2021*[41]	✓	✓	X	✓	✓	✓	✓	✓
**Reported type: Review**								
Bandali, 2019[42]	✓	✓	✓	✓	✓	✓	✓	X
Bausch, 1996[43]	✓	X	X	X	X	X	X	X
Biswas, 2015[44]	✓	✓	✓	✓	✓	✓	X	X
Chepkin, 2019[45]	✓	✓	✓	✓	✓	✓	✓	X
Harper, 1977[46]	✓	✓	X	X	✓	✓	X	X
Hinderaker, 2003[47]	✓	✓	X	X	X	✓	X	X
Hundley, 2001[48]	✓	X	X	X	X	✓	X	X
Kieltyka, 2012[49]	✓	X	✓	✓	✓	X	X	✓
Mo-suwan, 2009[50]	✓	✓	X	X	X	X	X	X
PMMRC, 2021[51]	✓	✓	✓	✓	✓	✓	✓	X
Rhoda, 2014[52]	✓	✓	X	✓	✓	X	✓	X
Sharma, 2022[53]	✓	✓	X	X	X	✓	✓	X
The National Center for Fatality Review and Prevention, 2021[54]	✓	✓	✓	✓	✓	✓	✓	X
Wilkins, 2015[55]	✓	✓	X	X	X	✓	X	X
**Reported type: Confidential inquiry**								

Amaral, 2011[56]	✓	✓	✓	✓	✓	✓	X	✓
Andersen, 1991[57]	✓	✓	X	X	✓	✓	X	✓
Cross-Sudworth, 2015[58]	✓	✓	X	X	✓	✓	✓	✓
De la Puente, 2002[59]	✓	✓	✓	X	✓	✓	X	X
Dekker, 2003[60]	✓	✓	X	X	✓	X	X	X
Draper, 2017[61]	✓	✓	X	X	✓	✓	✓	✓
Maternal and Child Health Research Consortium, 1999[62]	✓	✓	✓	✓	✓	✓	✓	✓
Mersey Region Working Party on Perinatal Mortality, 1982[63]	✓	✓	X	X	✓	✓	X	✓
Richardus, 2003[64]	✓	✓	X	X	✓	✓	X	✓
Stratulat, 2014[65]	✓	X	X	X	✓	X	✓	X
Tan, 1999[66]	✓	✓	X	X	✓	✓	X	✓
Tang, 2011[67]	✓	✓	X	X	✓	✓	X	✓
Vallejo, 1991[68]	✓	✓	X	X	X	✓	X	✓
D'Aloja, 2021*[41]	✓	✓	X	✓	✓	✓	✓	✓

A1: Data collection, A2: Case assessment of stillbirth, A3: Implementation of changes based on outputs, A4: Re-evaluation (i.e. a continuous cycle), R1: A collaboration between different specialities in obstetrics, midwifery, and pathology (i.e. the multidisciplinary team), R2: Examine reasons for cases for suboptimal care and/or avoidable factors, R3: Dissemination of key findings and learning points to all relevant clinical staff, C: Anonymous review
*D'Aloja, 2021 was described as both audit and confidential inquiry by the authors

Appendix 5: Included studies and the extracted data

Reference	Source of stillbirth data	Data collection method	Review tool or/and review method
Alderliesten, 2008[1]	- Perinatal deaths are reported to the investigator by fax	- The investigator fill in the structured case record form (CRF) on site after receiving a perinatal death notification	- All audit members are trained and the case summaries and clinical data (such as CTGs) are distributed 3 weeks prior to each audit session<br>- The review include an individual judgment of each case, using a structured judgment form, prior to the session<br>- A collective judgment of the perinatal audit panel follow after discussion during the audit session<br>- An independent chairman preside over all sessions<br>- The cause of death is recorded during the panel meetings, using three internationally accepted classification methods (Extended Wigglesworth classification, modified Aberdeen classification, fetal and infant classification)<br>- An overall review of the contribution of sub-standard care factors (SSCFs) in each case to the occurrence of perinatal death is determined and graded[$]<br>- The component of perinatal care (attributed to the mother and/or care provider) which was judged to be substandard (detection, therapy, logistics or communication) is recorded, as is the responsible care provider.<br>- Ten cases (7.3%) reviewed by the Amsterdam audit are allocated at random and presented to the external audit committee
Alyahya, 2021[2]	- Identified stillbirth among registered births in the five hospitals under study	- A death case review form with key details is filled from hospital notes and interviews from mothers, family members, relatives, and health worker	- The members of each death review committee (DRC) receive training<br>- Each DRC meet on a monthly basis to review all stillbirths and uses a specific form (mortality audit and action items form) to record the main cause of death; identify any critical delays and modifiable factors; identify the specific actions required to address the identified modifiable factors; and devise a follow-up plan
Amaral, 2011[56]	- Hospital records	- Data are collected from the medical and administrative records<br>- Clinical care provided in each case is compared with the standards defined in the Manuals on Emergencies and Humanized Healthcare (supervised by two research assistants and the principal investigator)	- Committee members identify potential delays in seeking, reaching or receiving care (presented as percentages) and a preventability score (PS) is proposed, based on the potential capacity of each intervention suggested to prevent the situation, ranging from 0 (none) to 5 (max) as judged by committee members in a participatory, consensus-driven process<br>- The mean PS is calculated from PS for each individual intervention, and the total per event is proposed as a product of the mean score of each intervention multiplied by the number of cases for which it was suggested
Aminu, 2017[3]	- Facility registers<br>- Delivery registers<br>- Surgical theatre registers<br>- Midwives' handover notes	- Data is collected using a specially designed data collection tool, informed by different countries' audit forms and consultation with 22 health care workers from the participating countries.<br>- Date of delivery, maternal sociodemographic characteristics, pregnancy details, obstetric and medical history, baby's characteristics, cause of death, and possible risk factors associated with the death are collected<br>- Other contextual information about the facilities are collected (e.g. signal function available, total deliveries)	- Health care professionals and the author review cases of stillbirth in batches within one month of death<br>- the completed forms from all participating hospitals are reviewed over several months, and at least one expert reviews each case. 1/4 of the sample is reviewed by a second reviewer<br>- data is entered into the data capture form using the Excel macro algorithms<br>- Standard of care is assessed using the grading system (0-2 only)*

Andersen, 1991[57]	- Medical records	- Data are entered into a questionnaire<br>- Detailed summary of each medical record is created	- The summaries and the questionnaires are sent to the members of each panel three weeks before the meetings<br>- The panel answer two questions: 1) would a different treatment have improved the infant's possibility of survival and 2) should a different treatment have been given<br>- Cases in which the panel answered "no" to one or both were allocated to "unavoidable deaths"<br>- Cases in which the panel answered "yes" to both were allocated to "potentially avoidable deaths"<br>- The panels are asked to state which suboptimal factor led to potentially avoidable deaths<br>- Suboptimal factors are categorised into suboptimal care in antenatal care or intrapartum care
Bandali, 2019[42]	- Perinatal Death Notification Form submitted to the facility Health Records Information Officer (HRIOs)<br>- Case notes from the hospitals	- Data extracted using National online forms (DHIS2) for perinatal death review reporting and case notes	- The Maternal and perinatal death surveillance and response (MPDSR) review meetings are held monthly to identify causes of death, avoidable causes, service delivery and quality gaps after the notification of perinatal death<br>- The review team fills out a review form which the HRIOs then submit as members of the review committee<br>- Perinatal death review actions are regularly tracked by the MPDSR committee, with feedback provided on the status of each recommendation.
Bausch, 1996[43]	- Fetal death certificates	- Data requests for information are sent from the Nebraska Medical Association to the medical records department of the hospital and to the physician singing the certificate of death	- The cases are numerically coded, and the medical information was mailed to reviewers<br>- Reviewers comment on aspects of prenatal care, intrapartum care, fetal data, cause of fetal death and if, in the reviewer's opinion, alteration of care received by the patient could have reduced the likelihood of fetal death
Berge, 1991[4]	- Antenatal charts<br>- Hospital records	Not stated	- Chart review<br>- Main cause of death and the avoidability of death are identified
Bhatt, 1989[5]	- Hospital records	- The unit concerned completes all papers on the deaths and submits the data to the committee, comprised of two members from the teaching staff along with the professor<br>- The committees raises questions and seek clarification on various points and if needed, the resident who managed the case will be called for additional information before it is filed.	- Six to eight meetings are conducted annually to discuss stillbirth<br>- The staff and residents are encouraged to freely express their opinions<br>- Ward nurses hold fortnightly meetings to discuss aspects of complicated obstetric cases
Biswas, 2015[44]	- Existing records<br>- Recall by visiting the health care providers in the facility who treated or managed the patient before death	- Using the facility death notification form and the death review form	- Senior staff nurses/family welfare visitors perform facility death notifications<br>- Facility death review meetings are conducting quarterly to analyse death cases and create action plans in the district and in upazilas
Bjellmo, 2019[6]	- Medical Birth Registry of Norway (MBRN)	- Case notes (open review) or a written detailed summary (blinded review)	- For open review, the two authors evaluate if the deliveries were in line with current Norwegian guidelines and if they could have been avoided if a planned caesarean delivery (CD) had been performed<br>- The course of the actual delivery is evaluated against both the guidelines relevant at the time of delivery and the current guideline and if the delivery was managed suboptimally<br>- To assess the suboptimal care, it is assessed subjectively by the two authors based on their clinical experience and the available written information

			- For blinded review, the two external examiners independently complete a questionnaire about the course of the delivery<br>- In this questionnaire, they are asked to indicate any deviations from the Norwegian guidelines and whether they considered the management of the delivery to be suboptimal or not<br>- They are also asked to predict whether they would expect the child to die or survive<br>- Regardless of this prediction, they are also asked to indicate whether the planned CD could have prevented death if the child had died<br>- The consensus meeting is conducted with all four authors
Chepkin, 2019[45]	- Deaths are notified within 7 days of the death and are then available within the Perinatal Mortality Review Tool (PMRT) to be reviewed using the PMRT	- Data is collected in the PMRT<br>- Relevant notes, statements, results of any follow-up investigations and other necessary information about the mother and her pregnancy<br>- Demographic details, pregnancy and medical history from the booking and antenatal information<br>- Parents' perspectives and any questions they raised<br>- A timeline of the events	- Once the data is collected, the PMRT presents the death details and provide *opening up* questions based on the information and responses provided for each question asked<br>- The questions consider the care provided and judgements about the quality of care provided<br>- The issues identified by the PMRT will be listed at the end of the review and the review group identifies the factors which contributed to this issue<br>- The review team selects contributory factors from the National Patient Safety Agency Contributory Factors Classification Framework<br>- The review team identifies whether that issue was likely to have contributed to the outcome for the baby and/or the mother<br>- The review group identifies the actions needed to improve care<br>- The review group are asked to consider and grade (0-3)* the quality of care provided for each of the following: 1) "the care provided to the mother and baby up to the point that the baby was confirmed as having died"; 2) "the care provided to the mother following confirmation of the death of her baby"
Copenhagen: WHO Regional Office for Europe, 2020[7]	- The State Statistical Office (SSO)<br>- Case notes	- Maternal characteristics and information on risk factors during pregnancy, care during pregnancy, social factors and quality of neonatal care is entered into a predesigned database	- Medical records are distributed to the review members<br>- The review members analyse the health care interventions given to the mothers, assess application of standards of care, possible delays in providing adequate care and any action points identified from the review<br>- The classifications and forms were adapted from the World health Organization (WHO), Making every baby count: audit and review of stillbirths and neonatal deaths and the United Nations International Children's Emergency Fund (UNICEF) capacity-building package
Cross-Sudworth, 2015[58]	Not stated	- Copies of relevant case notes are obtained<br>- A short risk assessment form is sent to mother's General Practitioner (GP) and to named community midwives to collect details of the social, psychological and medical risk factors	- Each panel has an independent chair and assisted by specialist midwives from the West Midlands Perinatal Institute<br>- The panel discuss and comment on the standard of care and avoidability of outcome based on CESDI criteria (0-3)*
D'Aloja, 2021[41]	- Each case is reported to the Regional Operational Unit (ROU) and National Operational Unit (NOU) within 48 hours through a web-based system	- During the first stage of the surveillance system (local audit), the SP1 anonymous paper form is filled in to summarise the mother and newborns clinical history, the complete care pathway, and the cause of death<br>- The SO online form describing the facility organisation to verify compliance with the national clinical standards requirements is filled in	- The perinatal mortality surveillance system (SPItOSS) operates via three organisational levels<br>- Local level conducts multiprofessional audits using the Significant Event Audit methodology<br>- Regional level has a ROU subgroup that conducts regional Confidential Enquiries once they are notified of perinatal deaths, where they review by prioritising the most preventable deaths in the following order: 1) intrapartum deaths, 2) neonatal deaths related to intrapartum foetal distress

			and subsequently other neonatal deaths, 3) antepartum deaths, starting from those occurring from 35 gestational weeks<br>- ROU assess the cause of death, identify clinical or organisational weaknesses in the care system, develop recommendations, and determine death avoidability<br>- The avoidability focuse on four criteria: 1) inappropriate care with avoidable outcome; 2) improvable care with unavoidable outcome; 3) appropriate care with unavoidable outcome; 4) unaccountable outcome<br>- National level revises and re-evaluates the regional Confidential Enquiries up to 100 cases (with the same priority protocol as regional level), and NOU also organises an annual national meeting with the Confidential Enquiries Regional Committee and the Confidential Enquiries National Committee experts to discuss the discordant cases and attain a shared evaluation
de Caunes, 1990[8]	- Telephone notification to audit process by any medical or paramedical staff member	- Within 24 hours after notification of death, the corpse, placenta cord are macroscopically examined; blood and cerebrospinal fluid are sampled for bacteriological culture; medical charts and hospital records are reviewed<br>- Medical and paramedical personnel and mothers are interviewed<br>- Once the data is compiled, a comprehensive report is sent for corrections to the obstetrician in charge of the delivery	- A meeting is held to discuss each case and complete a perinatal death certificate based on recommendations provided by the World Health Organization<br>- Cause of death and factors amenable through facility's practices are examined<br>- Avoidable deaths are discussed by conducting a critical review of the care involved while considering mothers' socioeconomic environment and perinatal resources available in the region<br>- With avoidable death that did not reach a consensus, an external reviewer is involved
De la Puente, 2002[59]	- Medical records<br>- the maternal health primary care providers via interviews	- Data is collected using medical records and interview<br>- Based on country of origin, women are categorised as born in Spain or immigrants.	- Obstetric suboptimal care is graded using the CESDI format (0-3)*<br>- Avoidable factors are identified using Mantel classification
Dekker, 2003[60]	- The National Obstetrics Registration Primary Care (Landelijke Verloskunde Registratie eerstelijn, LVR-1)<br>- National Obstetrics Registration Secondary Care (Landelijke Verloskunde Registratie tweedelijn, LVR-2)	- The status inquiry is made if data was missing or incomplete<br>- A file was created for each death	- The files are sent to the panel members, and they are asked to determine the cause of death using the Aberdeen classification, and determine if there had been suboptimal care, and to which extent this suboptimal care could have caused death<br>- Suboptimal factors is graded (0-3)$<br>- If there was no consensus when comparing individual grades, panel members can see other members' grades and reconsider their own score.<br>- If there was still no consensus after the second phase, a plenary meeting is organised to discuss the cases
Demise, 2015[9]	- Hospital's birth register	- Using a standardised data collection form data is abstracted from medical records and data is obtained by conducting interviews with health staff involved in the care of stillbirth, mothers and family members,<br>- The interviews are conducted using a pre-tested and structured questionnaire that incorporated a modified verbal autopsy instrument developed by the World Health Organization (WHO)	- The Audit Team determines the diagnosis and immediate cause of death, according to Wigglesworth classifications; whether or not the death was preventable; and if preventable, what specific avoidable factors contributed to the death<br>- Determining whether or not a death was avoidable rely upon serious consideration of information derived from multiple inputs and deliberation among members of the Audit Team<br>- When reviewing cases, the Audit Team take particular note of risk factors that are known to be associated with poor outcomes, and how these may have related to missed opportunities to provide standard care practices
Draper, 2017[61]	- The Mothers and Babies: Reducing Risk through Audits and Confidential Enquiries	- Copies of all relevant case notes are requested to be sent from the local Trust or Health Board teams	- Panel members receive the anonymised notes three weeks prior to the meeting

	across the UK (MBRRACE-UK) perinatal mortality surveillance system	to the MBRRACE-UK office (except Northern Ireland where they will gain consents from the family and redact records before sending them to the MBRRACE-UK office)<br>- Trusts and Health Boards provide details on notes that were unavailable and add any helpful how to locate them<br>- A research midwife prepares the notes for the inquiry panels in logical order, redacting identifiable or removing any irrelevant information and producing a summary	- A lead presenter is identified for each case from the panel and the lead is responsible for presenting at the meeting and completing a checklist prior to the meeting<br>- Panel meetings are chaired by one of three neutral chairs<br>- The panel's composition depend upon the type of cases being discussed<br>- A general discussion followed by a consensus opinion of the grade of quality of care (0-3)[&], relevance of grade of care to outcome (0-3)[+] and which aspects were suboptimal are noted (what, who) for each aspect of care (antenatal, during labour, at birth, post-natal and bereavement, post-mortem/placental histology)<br>- Overall quality is graded (0-2 only)* and a summary comments are given, and the panel evaluates if the overall grade relates to mother's health, care of the baby or both
Eksmyr, 1986[10]	- the Swedish Medical Birth Registry	- A summary is collated from the medical records	- The panel classifies each case into one of two categories, unavoidable and possibly avoidable deaths and specific details of avoidable factors with reference to the time period (antenatal or intrapartum) is stated<br>- The avoidable deaths are only referred to hospital care and not maternal or social factors<br>- The classification is based on the state of the art and time of delivery is blinded to the panel when making decision
El Amin, 2002[11]	- Registration of all births at the delivery wards	- Narratives on all cases of perinatal death are prepared in both Arabic and English.	- Audit meetings are held at 2-week intervals, and the last one is an open meeting presenting the results of the process and suggested measures for the future<br>- The narratives of cases for assessment and audit grading forms in English and Arabic are distributed to audit committee members two days before the audit meetings<br>- Based on clinical judgment, all cases are assessed individually by the auditors prior to the meetings<br>- A grading form on each case consisted of pre-structured questions: 1) do pre-pregnant risk factors or events during pregnancy indicate that the woman should have been delivered selectively before labour? 2) according to your judgement, considering the local resources, the death was associated with: optimal care (=1), probably acceptable care (=2), probably suboptimal care (=3) or suboptimal care (=4)<br>- Further comments specifying or discussing suboptimal conditions and care are requested<br>- During the meetings, all cases are presented by the PhD student, after which open discussions led to a consensus on the audit questions of the grading form<br>- An external audit panel of two Danish senior obstetricians who had knowledge about the local structure, organisation and resources in Sudan assessed all cases after the meetings<br>- In Sudan, no explicit criteria are followed, and therefore assessments reflect personal knowledge, experience, and culture
Eskes, 1993[12]	Not stated	Not stated	- The experts subdivide the cases into six groups: (1) unavoidable; (2) probably unavoidable; (3a) insufficient information; (3b) possibly avoidable; (4) probably avoidable; and (5) avoidable. In assessing (un)avoidability and

			the results were considered against the background of the most ideal circumstances and facilities in the time period concerned
Eskes, 2014[13]	- Perinatal Registry of The Netherlands (PRN)	- Stillbirths are registered in Perinatal Audit Registry of The Netherlands (PRN-Audit)<br>- Data are gathered from the medical records and registered with specific details needed to construct the narrative that will be used during the audit, including supplementary information such as professionals involved in the care process, diagnostics, policy decisions, actions (treatments, referrals) and antepartum risk selection with their time frames	- The basic document for the audit meeting is created from the PRN-Audit database<br>- Representatives of the professionals of the perinatal cooperation groups (PCG) analyse the cases, identify substandard care factors (SSF) in delivered care and/or organisation of care, identify the types of professionals involved and classify mortality according to three different systems (Wigglesworth/Hey, Modified ReCoDe, and Tulip classifications)<br>- Grades how likely SSF was the cause of death (none/unlikely, possible, (very) probable, unknown)<br>- Specific recommendations for improving the quality of care are formulated<br>- There is a chairperson in the meeting who is independent and from outside of the hospital
Essén, 2002[14]	- The national Swedish Medical Birth Register	- Narratives based on individual records are written for each birth solely from the data obtained	- Panellists are blind to the country of origin of each woman studied and work with the case narratives to identify suboptimal factors which were likely to have contributed to perinatal death<br>- Primary criteria (explicit and primarily evidence-based) for identifying suboptimal factors are adopted from the EuroNatal study of perinatal mortality in Europe<br>- Other suboptimal factors identified during the audit are categorised as secondary criteria. Suboptimal factors were assigned to one of three categories: maternal factors (which the mother did not realize were detrimental), medical care or communication<br>- To categorise perinatal deaths to ascertain which are potentially avoidable, a modified version of the Nordic-Baltic perinatal death classification scheme is used
Flenady, 2021[15]	- Queensland Perinatal Data Collection (QPDC), a legislated data collection of all births occurring in Queensland	- Data is collected from the Directors of Obstetrics (or equivalent) and/or Patient Safety and Quality Officer at the services where the deaths occurred and medical records	- Cases are summarised by the midwifery project coordinator and presented at the meeting<br>- Using the PSANZ guidelines tool, contributing factors were assigned to: 1) organisation/management (eg inadequate supervision of staff, lack of appropriate clinical management protocols, lack of communication between services), 2) personnel (eg staff factors relating to professional care and service provision), 3) accessing/engaging with care (e. no, infrequent or late booking for antenatal care, women decline treatment/advice)<br>- The contribution of each identified factor in the death was classified*
Fossen, 1999[16]	- Hospital evidence	- Data are collected from medical records	- The committee evaluate and assess to find suboptimal care regarding the identified stillbirth
Furst, 1989[17]	- the Government Central Bureau of Statistics	- Mother’s practice and hospital medical files that had satisfactory data entries on selected criteria are included and utilised for the audit<br>- A detailed interview with the practice doctors responsible for the mother's antenatal care before referral to hospital, hospital doctors responsible for the mother's hospital care, and with each mother are conducted	- A detailed chart review of the mother's practice and hospital medical files, as well as a detailed interview with relevant stakeholders (practice doctor, hospital doctor, and mothers) are audited

Govender, 2017[18]	- Hospital's Perinatal Problem Identification Programme (PPIP) database	- The 'Hands Up' Mortality and Morbidity Extraction Tool (HUMMET) form is used to summarise all cases	- Healthcare workers analyse the case and identify risk factors, avoidable factors, substandard care/missed opportunities, learning opportunities and actions to be taken regarding the patient and the health service<br>- The main factors contributing to each perinatal death are coded according to the PPIP and entered into the electronic PPIP database.
Han, 2018[19]	- Hospital Stillbirth Registry	- Data of patients' case records, summaries, results of investigations and post-mortem reports are obtained	- Using a prepared structured proforma, level of care, causative factors of stillbirths and whether the case was preventable are recorded by two assessors<br>- The factors of suboptimal care are graded (0-3)*<br>- Responsible personnel are determined for identified factors of suboptimal care<br>- The stillbirths are classified using the KK Hospital Stillbirth Classification
Harper, 1977[46]	- Death certificates	- Data is collected by abstracting medical records onto a 37-page form	- The abstracted chart data are reviewed by a panel of four physicians, independently<br>- Each reviewer is expected to define the factors which placed the mother and infant at risk and to identify the interventions which might prevent or alleviate problems in subsequent pregnancies by answering these questions: 1) what was the cause of death?, 2) was the death preventable?, 3) what were the factors that contributed to preventability?
Hinderaker, 2003[47]	- Prospective cohort of antenatal attendees	- Verbal autopsies<br>- Household survey interviews<br>- Hospital and medical records	- The causes of death is grouped into a functional classification developed by Wigglesworth et al. and modified by the International Collaborative Effort on BirthWeight, Plurality, Perinatal and Infant Mortality<br>- The underlying causes of death are grouped into seven functional categories where each group has common features requiring intervention at a specific time<br>- The two reviewers from the panel also assessed whether the deaths are "avoidable" and the factors that were suspected to be responsible for the avoidable deaths, are categorised into patient-oriented and health service provider-oriented
Hundley, 2001[48]	- Stillbirths identified in the Aberdeen trial in 1992-1993.	- EuroNatal questionnaire is used to extract required data from maternity, neonatal and autopsy records<br>- A narrative summary is written to provide details of chronological order	- The reviewers review the case histories independently and give a grading regarding suboptimal care before the panel meeting<br>- Panel meeting occurs after three weeks after receiving the case histories, observed and documented by the author<br>- One panel member presents the case and discussion follows after to grade the suboptimal care<br>- Suboptimal factors are classified into when it occurred (antenatal, intrapartum, or neonatal), then who was responsible for them (maternal/social, infrastructure/service organisation or care)<br>- Suboptimal factor is judged if it contributed to the stillbirth outcome using a grading system (unlikely, possibly, likely)<br>- Overall grade of the case is given (0-3)*
Kasengele, 2017[20]	- Case notes<br>- Routine data from the admission and delivery register	- data are collected using the structured checklist adopted from WHO	- A criteria-based clinical audit is conducted (using the criteria adopted from the Integrated Management of Pregnancy and Childbirth (IMPAC) programme<br>- Recommendations are developed to reduce the performance gaps.
Kieltyka, 2012[49]	- Obituaries<br>- Hospital records<br>- Louisiana Vital Events Registry	- Standardised data collection is performed using a web-based data system, BASINET	- Most of the LaFIMR Network CRTs meet quarterly<br>- CRT reviews 3-8 cases per two-hour session

		- BASINET automatically strips identifying information from case summaries and contains de-identified narrative case summaries, including information from maternal interviews	
Kirabira, 2020[21]	- Maternity registers<br>- Newborn admission register<br>- Hospital notes	- Data is abstract using an audit form<br>- A narrative summary is written for each case	- Each case is presented to the audit team for detailed discussion<br>- The information presented to the team allows for reviewing the cause of death and assigning a more precise cause of death by consensus<br>- Care provided is judged against existing local protocols and national guidelines.<br>- The quality of care provided at the hospital is also graded by consensus and classified into four categories*<br>- The overall grading of the care provided to the mother and baby, the identified specific areas of suboptimal care and the recommendations for improvement are documented<br>- The summary of each case included a list of the actions to be taken and during monthly meetings, implementation progress was compared with the action plan by the wider audit team.
Maaløe, 2016[22]	- Admission, delivery, and theatre registers<br>- Case files	- Data is extracted into a structured entry form based on the pre-selected audit criteria	- Case files of stillbirths are reviewed using the criteria of realistic best quality of care
Maternal and Child Health Research Consortium, 1999[62]	- The Rapid Report Form<br>- National collection of mortality statistics by the Office for National Statistics (ONS)	- Case notes identifying/documenting risk factors and management during antepartum and intrapartum periods	- Confidential inquiry is conducted by a regional multidisciplinary panel<br>- The panels reviews all aspects and record suboptimal care (notable factor).<br>- For each notable factor, panel assigns when, what and when category; grade of the standard of care (0-3)*; grade of the relevance to death (0-3)$^{+}$<br>- Panel assigns cause of death according to the pathophysiological classification by Wigglesworth, the fetal and neonatal classification, the revised Aberdeen Obstetric classification and an overall grade relating the care to the final outcome*.<br>- Panel comments on the completeness of the record (case notes).
Mbaruku, 2009[23]	- Interview<br>- Hospital records	- A detailed questionnaire is filled in by one of the authors	- The reviewers audit using the 3-delays methodology which classifies delays as follows: 1) first delay refers to the time spent at home before a decision is made to seek health care; 2) the second delay refers to the inability to get to a health facility after having made the decision to go there (due to problems with transport, money, or poor roads); and 3) the third delay refers to the time spent waiting for adequate management after arrival at a health facility<br>- The reviewers consider mismanagement or incorrect treatment as a third delay<br>- A process audit is undertaken, whereby actual practice is compared with standard practice based on evidence or expert consensus
Mdoe, 2022[24]	- Patient's case notes	- Data were collected using a designated case review form (CRF) from the patient's case notes	- The CRF is reviewed by a clinician who commented on the possible cause of death and a likely contributing factor(s) based on the reviewed case notes<br>- The research team includes the obstetrician, pediatrician, and senior midwife and reviews the comments made by a clinician<br>- The agreed completed CRF is entered into the database for analysis
Mersey Region Working Party on Perinatal	- Notification of death by telephone to the inquiry office by a senior nursing officer from each maternity unit or each specialist in community medicine	- After each notification, a full-time member of the working party visits the maternity unit within 5 days of death<br>- A questionnaire is completed using the case notes	- The assessors (panel) assess which factors were avoidable and is grouped into the following three groups – obstetric; paediatric; and maternal/social<br>- The Scottish perinatal mortality survey classification of death is used to classify cause of death

Mortality, 1982[63]		- The professional staff concerned in the care of each case are interviewed<br>- The mother's GP is contacted by telephone and, if necessary, visited.<br>- The mother is interviewed by another member of the working party and the interview helps filling out the questionnaire form	
Miranda, 1996[25]	- Patient records	- Information obtained from patient records	- The committee carries out a detailed analysis of each case<br>- The committee assess if the suboptimal care was relevant to the outcome
Mo-suwan, 2009[50]	- The Prospective Cohort Study of Thai Children (PCTC)	- An interview to collect baseline data of pregnant women and their families at 28-36 weeks gestation<br>- Home visits to these women are carried out weekly from 36 weeks onwards<br>- The delivery outcome was retrieved from the hospital records<br>- Interviews with parents and caregivers after the death are also collected	- Causes of death are defined based on Wigglesworths classification<br>- The reviewers are asked to conclude whether death of that case was preventable
Moawad, 1990[26]	- Clinical records<br>- Pathologic diagnosis	- Data are entered on a specially designed form<br>- A narrative case summary is prepared	- Audit meetings are held in the hospital where the death had occurred<br>- Avoidable death is identified through clear consensus
Musafili, 2017[27]	Not stated, assumed hosptial records	- Using questionnaires, hospital data is abstracted and information is obtained from interviews with mothers and healthcare providers<br>- Narratives summarising the information collated from the different sources are prepared for each case by the main investigator	- The main investigator introduce the study objectives and procedures at the first meeting and present all cases of perinatal deaths discussed in each session, which was scheduled according to the members' availability<br>- These presentations are based on individual narratives and are followed by open discussions to identify the causes of the deaths, the underlying factors, and any potentially avoidable deaths<br>- The discussions aim to evaluate whether the delay had possibly or likely contributed to the fatal outcome<br>- The quality of care provided to mothers and neonates are judged against evidence-based practices, as expressed in local guidelines and protocols or the scientific literature
Omwodo, 2020[28]	Not stated, but assumed hospital records	Not stated	- Presentations are given for each case<br>- Open discussions by the panel to identify the cause of death, the underlying factors, any potentially avoidable deaths and build recommendations for improved care
Pattinson, 1995[29]	Not stated	Not stated	- All stillbirth are classified according to the primary obstetric and neonatal causes of death<br>- Potentially avoidable factors which could have contributed to the death are identified, classified and graded<br>- The avoidable factors are classified into patient-oriented, administrative and medical management-related groups<br>- The patient-oriented category is further divided into an inappropriate response to a complication, non-or late attendance at antenatal clinics, and criminal intervention during the pregnancy<br>- The medical management category is further divided into honest errors, oversight and a gross deviation from accepted practice.<br>- Avoidable factors are classified into two grades: grade 1 - an action which, if avoided or altered, could possibly have modified the outcome, and grade 2 -

			actions which, if avoided, would probably have altered the outcome, in which case the baby might have survived<br>- All cases are presented by a doctor who is specifically assigned the presentation task to allow the problem to be discussed freely<br>- The final categorisation of avoidable factors are made by one researcher after the assessment of the case notes to ensure consistency
PMMRC, 2021[51]	- Compilation of data submitted by Lead Maternity Carers, local coordinators, the Ministry of Health and Births, Deaths, and Marriages registry	- Local coordinators oversee the collection of data<br>- Data is submitted to the New Zealand Mortality Review Data Group at the University of Otago<br>- Coordinators are required to submit rapid reporting forms within 48 hours of death<br>- Two forms: one containing information on the mother and one form containing information on the baby	- Local coordinator initiates local clinical reviews, assign cause of death using Perinatal Society of Australia and New Zealand (PSANZ) Perinatal Death Classification, contributory factors, potentially avoidable deaths<br>- A multidisciplinary team completes the Perinatal and Maternal Mortality Review Committee (PMMRC) classification form<br>- Local coordinator submits the post-mortem and histology reports with the classification form
Raman, 2015[30]	Not stated	- A clinician enter the demographic information for each perinatal death onto the datasheet and information on modifiable factors is completed in discussion with a senior doctor	- Death is categorised using the Perinatal Society of Australia and New Zealand perinatal mortality classification<br>- Modifiable factors related to each death is classified according to the three delays model
Rhoda, 2014[52]	- Hospital data<br>- Birth register in the labour ward	- Data Capturing Form is used to insert basic demographic data and primary obstetric cause of death	- The causes of stillbirths is divided into primary obstetric causes and the final neonatal causes<br>- Each case is categorised as an avoidable or unavoidable death<br>- For avoidable deaths, it is further categorised into the patient, medical-personnel- or administrative-related deaths
Richardus, 2003[64]	- Student Center Perinatal Epidemiology (SPE) in Flanders<br>- Danish Brith Register<br>- Confidential Enquiry into Stillbirths and Deaths in Infancy (CESDI)<br>- Medical Birth Register<br>- 2nd Greek National Perinatal Survey<br>- Hospital case records and midwife case records, Medical Birth Registry of Norway<br>- Aberdeen Maternal and Neonatal Databank, Hospital registers: delivery-, case record- and pathology register<br>- Regional Medical Register "Perinatal Revision South"	- Explicit criteria for standards of care are defined by the Euronatal Working Group and are incorporated into a questionnaire, which is completed in the country of origin<br>- The excerpt, narrative summary and questionnaire are produced from questionnaire data by the co-ordination team	- A grading system is adapted from the Confidential Enquiry into Stillbirths and Deaths in Infancy to define suboptimal care<br>- Each case is blinded to the country and distributed randomly to one subpanel<br>- Each subpanel member receives a copy of the questionnaire, the narrative summary and a sheet that identified a lack of compliance with the explicit audit criteria<br>- After reviewing these, the panel member decides whether there were instances of suboptimal care and any such instances are listed on a standard form to mark timing and responsible individual/organisation
Sauvegrain, 2020[31]	- Maternity and neonatal unit notification	- Data is abstracted from medical records using a pre-tested structured instrument<br>- Maternal interviews are conducted	- For each case, the reviewers identify suboptimal care factors and provide a citation for the guideline or standard or an explanation<br>- The reviewers judge whether the factors related to the death and whether acting on them could have prevented it<br>- The reviewers also evaluate whether the information was complete and adequate for their assessment<br>- The reviewers meet as a panel to harmonise definitions and evaluations and to discuss cases flagged for group review
Sharma, 2022[53]	- Delivery registers<br>- Hospital records	- The information is collected using a pre-structured questionnaire according to the guidance in WHO, Making Every Baby count.	- The qualitative (identify modifiable or associated factors to stillbirth) and quantitative analysis is carried out every month

		- Interviews with patients and husbands/family members is conducted wherever possible	- Some cases are selected for detailed discussion in the monthly perinatal meeting.
Sterpu, 2020[32]	- Obstetric record system Obstetrix	- The data are collected from the obstetric record system Obstetrix, TakeCare, Swedish Medical Birth Register and hospital records	- The review team assess cause of death, the proportion of preventable/non-preventable stillbirth and the level of delay (patient/caregiver) and details, demographic details, pregnancy characteristics and fetal outcomes
Stratulat, 2014[65]	Not stated	Not stated	- Cases are analysed and the causes of death are examined using the ReCoDe classification<br>- Cases were graded according to the presence of substandard care, and whether that was considered as contributing to the adverse outcome
Supratikto, 2002[33]	- Report to the village midwife or district health office	- The village midwife conducts post mortem and obtain medical record<br>- All forms checked by a senior midwife or a doctor and sent to the district health office	- The village midwife presents the background and the chronology of events and the review participants consider the case, relying on the expertise of an obstetrician or paediatrician from the district hospital to guide discussion on clinical case management<br>- On the basis of the contributing factors identified during the meeting, the audit team at the district level designs management initiatives, proposes additional training, and recommends changes in clinical protocol and policy<br>- The findings and recommendations have discussed at the start of the next audit meeting in order to check on progress made in resolving the problems previously uncovered
Tan, 1999[66]	Not stated	Not stated	- Assessors first work independently using a structured assessment protocol<br>- After collating the assessment forms, the coordinator arrange a meeting of the entire panel for discussion and a panel consensus is reached for each case<br>- Factors of sub-optimal care to clinical practice, staffing, structure, equipment and patient or her family are identified in each case and they are graded*
Tang, 2011[67]	Not stated. Assumed hospital records	- Data is recorded on the pro forma prospectively following the event and checked with the hospital records	- The panel review the pro formas anonymously and case notes are consulted where additional information is required<br>- The management of each stillbirth is allocated to one of the four standards of care categories established by CESDI
The National Center for Fatality Review and Prevention, 2021[54]	- Identified deaths to Fetal and Infant Morality Review (FIMR) staff<br>- Hospital/birthing center admission and discharge logs<br>- Obituary columns, through communication with existing local programs, agencies, hospital/birthing center bereavement nurses or counsellors<br>- Local doula providers<br>- Funeral directors<br>- Hospital medical records staff<br>- Medical examiner or coroners's offices,<br>- Emergency medical transport service teams<br>- Community health workers	- Summaries of 3-5 cases from source of data, related social services records, and the parental/family interview, prepared by FIMR staff before the meeting.	- FIMR staff coordinates and schedule meeting with the Case Review Team (CRT)<br>- The multidisciplinary CRT reviews the barriers to care, trends in service delivery and action points improve policies and services<br>- The CRT analyses the information and summarise findings to create recommendation
Vallejo, 1991[68]	- Texas department of health<br>- Death certificates	- Data of birth and death certificate combined with the maternal/infant clinical notes contained in the hospital or midwife records using the perinatal morality survey tool	- Records review
Vallely, 2020[34]	- Severe adverse events (SAE) reports	- Using a study-specific SAE form, data is collected for the mother's general health, pregnancy risk	- Two members conduct the initial analysis and coding of each case, independently

		factors, and the circumstances around the event including prenatal, labour, and birth events, gestational age at birth, birth weight, and final outcome	- 10% of the cases are randomly selected, assessed, and coded independently by two obstetricians
van Diem, 2010[35]	Not stated, assumed hospital records	- Data are entered and described on an anonymous narrative form	- Using the SPSS function "random selection" a subgroup of cases is selected using the case numbers as variables for selection<br>- Blinded to each other, the auditors assess these cases once individually and later at a group discussion<br>- For the classification of the cause of perinatal death, the modified Aberdeen Classification, the Extended Wigglesworth Classification and the Tulip classification is used<br>- Substandard factors (SSF) is graded as: no SSF identified and one or more SSF identified<br>- The avoidability is graded[+]
van Diem, 2012[36]	- Hospital/unit records<br>- Narratives of care team	- Anonymous narratives are the basic documents used in the perinatal audit meetings and it includes the cause of death classified by the local core group	- Using the narrative, SSF are identified during unit based perinatal audit meetings<br>- The audit instrument is based on a root cause analysis as 6 What Questions: 1) what happened and which caregivers were involved, 2) what were the circumstances in which the SSF occurred, 3) what made the SSF occur, 4) what is the relation between the SSF and death, 5) what are the conclusions, 6) what needs to be done to prevent the SSF from occurring again<br>- Members of the project team chair the meetings until other independent chairpersons is found
Ward, 1995[37]	Not stated, assumed hospital records	- Scrutinised case records	- The primary obstetric cause of the sequence of events leading to each death is classified using the Whitfield classification<br>- Each death is assessed for potentially avoidable factors; if found, were graded, categorised and subdivided.
Westergaard, 1997[38]	Not stated	- Summaries of pregnancy, delivery and foetus are built by reviewing clinical records and discharge letters	- Each review member is given a summary of death and asked to answer three questions: 1) could the fetus already have been dead before admission (1=possibly, 2=less likely, 3=not possible); 2) were dispositions (surveillance and interventions) during delivery suboptimal (1=to a high degree, 2=to a less degree, 3=no) ; 3) could an improved effort during delivery have prevented the intrapartum death (1=yes, certainly, 2=yes, possibly, 3=no, probably not)<br>- Reviewers are asked if anything could have done differently and to provide any additional comments
Wilkins, 2015[55]	- Hospital birth registry<br>- Maternal records	- Data is collected and entered directly into a spreadsheet, adapted from the WHO Southeast Asia Regional Office stillbirth record form	- The simplified Cause of Death and Associated Conditions (CODAC) classification system, developed specifically for use in low-income settings, is used to classify cause of death<br>- The quality of care is assessed based on the reviewers' knowledge of the available resources and staff training from extensive time spent as an obstetrician in the hospital (no formal tool)
Wilkinson, 1997[39]	- Case records<br>- Maternity register<br>- The ward death notification book<br>- The mortuary admission book	- Data are collected daily by carefully reviewing case records, discussing the case with the staff involved and interviewing the mother	- The perinatal deaths are assessed and all avoidable deaths are classified

Wolleswinkel-van den Bosch, 2002[40]	- Gynecology/obstetrics departments and labour ward registers	- Medical records of the identified cases are obtained from the medical archives of the hospitals or the midwives and these records are summarised using a structured questionnaire<br>- A case abstract is written in order to better represent the chronology of events during perinatal care<br>- Doctors and midwives are asked to verify the data as summarised by the investigators.	- For each case, the panel members first note deviations from the criteria and their relationship with perinatal death (unlikely, possibly or probably' related to perinatal death) on a score sheet<br>- The panel members then gave a final score, based on all substandard factors identified[$]<br>- The panel members could also indicate whether sufficient information was available to give a score<br>- A plenary panel meeting is held if the consensus is not reached

*0 - No suboptimal care. 1 - Suboptimal care, but different management would have made no difference to the outcome. 2 - Suboptimal care - different management might have made a difference to the outcome. 3 - Suboptimal care - different management would reasonably have been expected to have made a difference to the outcome
+0 - Not relevant. 1 - Possibly relevant. 2 - Probably relevant. 3 - Almost certainly relevant.
&0 - No suboptimal care. 1 - Minor suboptimal care. 2 - Significant suboptimal care. 3 - Major suboptimal care.
$0 - No suboptimal factors. 1 - one of more suboptimal factor, unlikely that this caused perinatal death. 2 - one or more suboptimal factor, possible this caused the death. 3 - one or more suboptimal factor, probable this caused the death.

Appendix 6: Quality assessment of the included studies

Table A7. Detailed quality assessment of the included studies

	Quality assessment criteria								
**First author and year**	**1**	**2**	**3**	**4**	**5**	**6**	**7**	**8**	**9**
Alderliesten, 2008[1]	Y	U	U	Y	Y	N	Y	Y	NA
Alyahya, 2021[2]	Y	Y	Y	Y	N	Y	Y	Y	NA
Amaral, 2011[56]	Y	Y	Y	Y	Y	Y	N	Y	NA
Aminu, 2017[3]	Y	Y	Y	Y	Y	Y	N	Y	NA
Andersen, 1991[57]	Y	Y	Y	Y	N	Y	N	Y	Y
Bandali, 2019[42]	Y	U	Y	N	N	N	Y	Y	NA
Bausch, 1996[43]	Y	Y	U	Y	N	N	N	N	NA
Berge, 1991[4]	Y	U	Y	Y	N	N	N	Y	Y
Bhatt, 1989[5]	N	U	Y	U	N	N	N	Y	NA
Biswas, 2015[44]	Y	U	U	U	N	N	Y	Y	NA
Bjellmo, 2019[6]	Y	Y	Y	Y	Y	N	N	N	Y
Chepkin, 2019[45]	Y	Y	Y	N	Y	Y	N	Y	NA
Copenhagen: WHO Regional Office for Europe, 2020[7]	Y	Y	Y	Y	Y	N	N	NA	Y
Cross-Sudworth, 2015[58]	Y	Y	Y	Y	Y	Y	N	Y	NA
D'Aloja, 2021[41]	Y	Y	Y	N	N	N	N	Y	NA
de Caunes, 1990[8]	Y	Y	Y	Y	N	N	N	N	NA
De la Puente, 2002[59]	Y	Y	Y	Y	N	Y	Y	NA	NA
Dekker, 2003[60]	Y	Y	Y	Y	Y	Y	N	Y	NA
Demise, 2015[9]	Y	Y	Y	Y	Y	Y	Y	Y	NA
Draper, 2017[61]	Y	Y	Y	Y	Y	Y	N	Y	Y
Eksmyr, 1986[10]	Y	U	Y	Y	N	Y	N	Y	NA
El Amin, 2002[11]	Y	Y	Y	N	Y	Y	N	Y	Y
Eskes, 1993[12]	Y	Y	U	Y	N	Y	N	Y	NA
Eskes, 2014[13]	Y	Y	Y	Y	U	Y	N	Y	Y
Essén, 2002[14]	Y	U	Y	Y	Y	Y	N	NA	Y
Flenady, 2021[15]	Y	Y	Y	Y	Y	Y	N	N	Y
Fossen, 1999[16]	Y	U	Y	Y	N	Y	N	Y	NA
Furst, 1989[17]	Y	N	Y	Y	N	N	N	Y	NA
Govender, 2017[18]	Y	Y	Y	Y	Y	Y	Y	Y	NA
Han, 2018[19]	Y	Y	Y	Y	Y	Y	N	Y	NA
Harper, 1977[46]	Y	N	Y	Y	Y	Y	N	NA	Y
Hinderaker, 2003[47]	Y	Y	Y	Y	Y	Y	N	Y	Y
Hundley, 2001[48]	Y	U	NA	NA	Y	Y	N	Y	NA
Kasengele, 2017[20]	Y	Y	Y	Y	N	N	Y	Y	NA
Kieltyka, 2012[49]	Y	Y	Y	N	N	N	N	Y	NA
Kirabira, 2020[21]	Y	Y	Y	N	Y	Y	Y	Y	Y
Maaløe, 2016[22]	Y	Y	Y	Y	Y	N	N	Y	Y
Maternal and Child Health Research Consortium, 1999[62]	Y	Y	U	N	Y	Y	Y	Y	NA
Mbaruku, 2009[23]	Y	Y	Y	Y	N	Y	N	Y	N
Mdoe, 2022[24]	Y	Y	Y	Y	Y	Y	N	Y	NA
Mersey Region Working Party on Perinatal Mortality, 1982[63]	Y	U	U	Y	N	Y	N	Y	NA
Miranda, 1996[25]	Y	Y	Y	Y	N	Y	N	Y	NA
Mo-suwan, 2009[50]	Y	Y	Y	Y	Y	N	N	Y	Y
Moawad, 1990[26]	Y	Y	Y	Y	N	Y	N	Y	NA
Musafili, 2017[27]	Y	Y	Y	Y	Y	Y	N	Y	NA
Omwodo, 2020[28]	Y	Y	Y	Y	Y	N	N	Y	NA
Pattinson, 1995[29]	Y	Y	U	U	N	Y	Y	N	NA
PMMRC, 2021[51]	Y	Y	Y	Y	Y	Y	N	N	Y
Raman, 2015[30]	Y	N	U	Y	Y	Y	N	Y	Y
Rhoda, 2014[52]	Y	U	Y	Y	N	N	N	NA	NA
Richardus, 2003[64]	Y	Y	Y	Y	N	N	N	Y	Y
Sauvegrain, 2020[31]	Y	Y	Y	Y	Y	N	N	Y	Y
Sharma, 2022[53]	Y	Y	Y	Y	Y	Y	Y	Y	NA
Sterpu, 2020[32]	Y	Y	Y	Y	Y	Y	N	Y	Y
Stratulat, 2014[65]	Y	U	U	Y	N	N	Y	NA	NA
Supratikto, 2002[33]	N	U	Y	U	N	N	N	Y	NA
Tan, 1999[66]	Y	Y	Y	Y	Y	Y	N	Y	NA
Tang, 2011[67]	Y	U	U	Y	N	Y	N	Y	Y
The National Center for Fatality Review and Prevention, 2021[54]	Y	U	Y	U	N	N	N	Y	NA
Vallejo, 1991[68]	Y	Y	Y	Y	Y	Y	N	Y	NA
Vallely, 2020[34]	Y	Y	Y	Y	Y	Y	N	Y	NA
van Diem, 2010[35]	Y	U	Y	Y	N	N	N	NA	Y
van Diem, 2012[36]	Y	Y	Y	Y	Y	Y	Y	Y	NA
Ward, 1995[37]	Y	Y	Y	Y	N	Y	N	Y	Y
Westergaard, 1997[38]	Y	Y	Y	Y	Y	Y	N	NA	Y

Wilkins, 2015[55]	Y	Y	Y	Y	Y	Y	N	Y	NA
Wilkinson, 1997[39]	Y	U	Y	Y	N	N	N	Y	NA
Wolleswinkel-van den Bosch, 2002[40]	Y	Y	Y	Y	N	Y	N	Y	Y

1. Were there clear criteria for inclusion in the review?
2. Was the condition (stillbirth) measured in a standard, reliable way for all fetus included in the review?
3. Were valid methods used for identification of the condition (stillbirth) for all fetus included in the review?
4. Did the review have consecutive inclusion of stillbirth cases?
5. Was there clear reporting of the demographics of the mothers who had a stillbirth in the review?
6. Was there clear reporting of "quality of clinical care" of the stillbirth cases?
7. Were the outcomes or follow-up results of having a review process clearly reported?
8. Was there clear reporting of the presenting site(s)/clinic(s) demographic information?
9. Was statistical analysis appropriate?
Y: Yes, N: No, U: Unknown, NA: Not applicable

Figure A7. Summary graph of quality assessment of the included studies

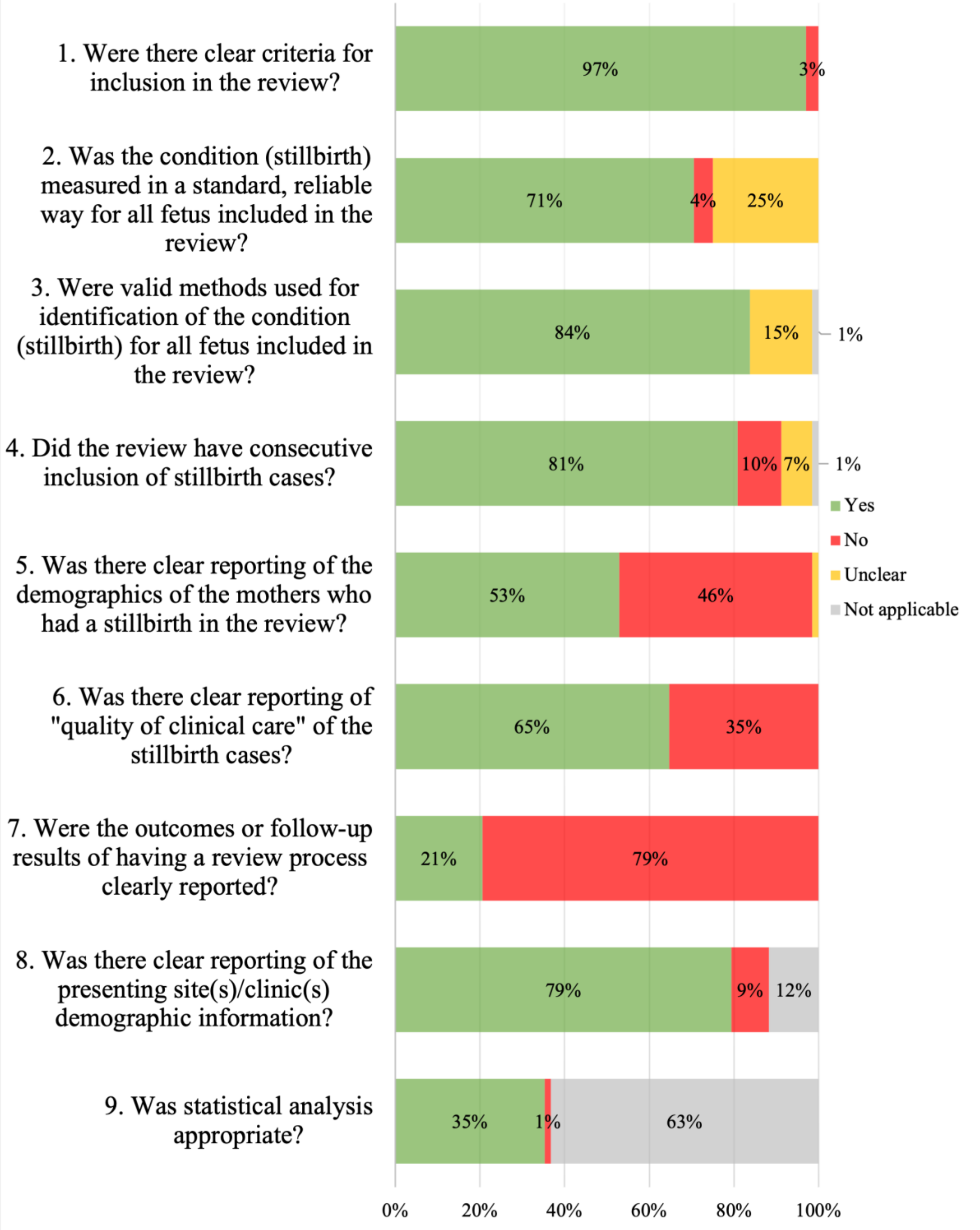

Note: Out of 68 included studies, each green bar states the proportion of studies that fulfilled the respective risk of bias question, red states those that did not, yellow states that were unclear and grey that were not applicable.

References

1 Alderliesten ME, van Lith JMM, Bleker OP, *et al.* Design and evaluation of a regional perinatal audit. *Eur J Obstet Gynecol Reprod Biol* 2008; **137**: 141–5.

2 Alyahya MS, Khader YS, Al-Sheyab NA, Shattnawi KK, Altal OF, Batieha A. Modifiable Factors and Delays Associated with Neonatal Deaths and Stillbirths in Jordan: Findings from Facility-Based Neonatal Death and Stillbirth Audits. *Am J Perinatol* 2021. DOI:https://dx.doi.org/10.1055/s-0041-1730434.

3 Aminu M. Cause of and Factors Contributing to Stillbirth in Sub-Saharan Africa. PQDT - Glob. 2017. https://www.proquest.com/dissertations-theses/cause-factors-contributing-stillbirth-sub-saharan/docview/2424363803/se-2?accountid=13042.

4 Berge LN, Rasmussen S, Dahl LB. Evaluation of fetal and neonatal mortality at the University Hospital of Tromso Norway, from 1976 to 1989. *Acta Obstet Gynecol Scand* 1991; **70**: 275–82.

5 Bhatt R V. Professional responsibility in maternity care: Role of medical audit. *Int J Gynecol Obstet* 1989; **30**: 47–50.

6 Bjellmo S, Hjelle S, Krebs L, Magnussen E, Vik T. Adherence to guidelines and suboptimal practice in term breech delivery with perinatal death- a population-based case-control study in Norway. *BMC Pregnancy Childbirth* 2019; **19**: N.PAG-N.PAG.

7 Copenhagen: WHO Regional Office For Europe. Perinatal mortality audit: North Macedonia 2019. World Health Organization, 2020.

8 De Caunes F, Alexander GR, Berchel C, Guengant J-P, Papiernik E. The Guadeloupean perinatal mortality audit: Process, results, and implications. *Am J Prev Med* 1990; **6**: 339–45.

9 Demise A, Gebrehiwot Y, Worku B, Spector JM. Prospective Audit of Avoidable Factors in Institutional Stillbirths and Early Neonatal Deaths at Tikur Anbessa Hospital in Addis Ababa, Ethiopia. *Afr J Reprod Health* 2015; **19**: 78–86.

10 Eksmyr R, Larssen KE, Bakketeig LS, *et al.* Perinatal mortality in a Swedish county 1973-1978. Time trends revealed by perinatal audit. *Acta Paediatr Scand* 1986; **75**: 17–23.

11 El Amin S, Langhoff-Roos J, Bødker B, *et al.* Introducing qualitative perinatal audit in a tertiary hospital in Sudan. *Heal Policy Plan* 2002; **17**: 296–303.

12 Eskes M, Van Alten D, Treffers PE. The Wormerveer study; perinatal mortality and non-optimal management in a practice of independent midwives. *Eur J Obstet Gynecol Reprod Biol* 1993; **51**: 91–5.

13 Eskes M, Waelput AJM, Erwich JJHM, *et al.* Term perinatal mortality audit in the Netherlands 2010-2012: A population-based cohort study. *BMJ Open* 2014; **4**: e005652.

14 Essen B, Bodker B, Sjoberg NO, *et al.* Are some perinatal deaths in immigrant groups linked to suboptimal perinatal care services?. *BJOG* 2002; **109**: 677–82.

15 Flenady V, Kettle I, Laporte J, *et al.* Making every birth count: Outcomes of a perinatal mortality audit program. *Aust N Z J Obstet Gynaecol* 2021; **61**: 540–7.

16 Fossen D, Silberg IE. Perinatal deaths in the county of Ostfold 1989-97. *Tidsskr den Nor laegeforening* 1999; **119**: 1272–5.

17 Furst AL, Shamba E. Methodological issues associated with an audit of stillbirth in a family medicine practice in Israel. *Fam Pract* 1989; **6**: 177–81.

18 Govender I. Auditing stillbirths at Lower Umfolozi War Memorial Regional Hospital: A 12-month review. *S Afr Med J* 2017; **107**: 1121–6.

19 Han JY, Huang EY, Tan KH. Targeted patient education-A critical step toward reducing stillbirth rates. *Perinatology* 2018; **19**: 41–9.

20 Kasengele CT, Mowa R, Katai C, *et al.* Factors contributing to intrapartum stillbirth: a criteria-based audit to support midwifery practice in Zambia. *African J Midwifery Women's Heal* 2017; **11**: 67–71.

21 Kirabira VN, Byaruhanga R, Okong P, Dewez JE, Van Den Broek N, Aminu M. Prospective study to explore changes in quality of care and perinatal outcomes after implementation of perinatal death audit in Uganda. *BMJ Open* 2020; **10**: e027504.

22 Maaloe N, Housseine N, Bygbjerg IC, *et al.* Quality of intrapartum care at an East African referral hospital: A case-control study of stillbirths. *BJOG An Int J Obstet Gynaecol* 2016; **123**: 124.

23 Mbaruku G, van Roosmalen J, Kimondo I, Bilango F, Bergstrom S. Perinatal audit using the 3-delays model in western Tanzania. *Int J Gynecol Obstet* 2009; **106**: 85–8.

24 Mdoe P, Katengu S, Guga G, *et al.* Perinatal mortality audit in a rural referral hospital in Tanzania to inform future interventions: A descriptive study. *PLoS One* 2022; **17**: e0264904.

25 Miranda JA, Herruzo AJ, Mozas J, *et al.* Influence of obstetric and perinatal care on perinatal mortality. *Eur J Obstet Gynecol Reprod Biol* 1996; **67**: 103–7.

26 Moawad AH, Lee KS, Fisher DE, Ferguson R, Phillippe M. A model for the prospective analysis of perinatal deaths in a perinatal network. *Am J Obstet Gynecol* 1990; **162**: 15–22.

27 Musafili A, Persson L-Å, Baribwira C, Påfs J, Mulindwa PA, Essén B. Case review of perinatal deaths at hospitals in Kigali, Rwanda: perinatal audit with application of a three-delays analysis. *BMC Pregnancy Childbirth* 2017; **17**: 1–13.
28 Omwodo K. A qualitative perinatal audit at a rural health facility in Eldoret, Kenya. 2020; **7**. http://imsear.searo.who.int/handle/123456789/202097.
29 Pattinson RC, Makin JD, Shaw A, Delport SD. The value of incorporating avoidable factors into perinatal audits. *South African Med J* 1995; **85**: 145–7.
30 Raman S, Iljadica A, Gyaneshwar R, Taito R, Fong J. Improving maternal and child health systems in Fiji through a perinatal mortality audit. *Int J Gynecol Obstet* 2015; **129**: 165–8.
31 Sauvegrain P, Carayol M, Piedvache A, *et al.* Understanding high rates of stillbirth and neonatal death in a disadvantaged, high-migrant district in France: A perinatal audit. *Acta Obstet Gynecol Scand* 2020; **99**: 1163–73.
32 Sterpu I, Bolk J, Perers Öberg S, Hulthén Varli I, Wiberg Itzel E. Could a multidisciplinary regional audit identify avoidable factors and delays that contribute to stillbirths? A retrospective cohort study. *BMC Pregnancy Childbirth* 2020; **20**: N.PAG-N.PAG.
33 Supratikto G, ME W, Achadi E, Cohen S, Ronsmans C. A district-based audit of the causes and circumstances of maternal deaths in South Kalimantan, Indonesia. *Bull World Health Organ* 2002; **80**: 228.
34 Vallely LM, Smith R, Bolnga JW, *et al.* Perinatal death audit and classification of stillbirths in two provinces in Papua New Guinea: a retrospective analysis. *Int J Gynecol Obstet* 2020; **153**: 160–8.
35 Van Diem M, De Reu P, Eskes M, *et al.* National perinatal audit, a feasible initiative for the Netherlands!? A validation study. *Acta Obstet Gynecol Scand* 2010; **89**: 1168–73.
36 van Diem MT, Timmer A, Bergman KA, *et al.* The implementation of unit-based perinatal mortality audit in perinatal cooperation units in the northern region of the Netherlands. *BMC Health Serv Res* 2012; **12**: 195.
37 Ward HRG, Howarth GR, Jennings OJN, Pattinson RC. Audit incorporating avoidalbility and appropriate intervention can significantly decrease perinatal mortality. *South African Med J* 1995; **85**: 147–50.
38 Westergaard HB, Langhoff-Roos J, Larsen S, Borch-Christensen H, Lindmark G. Intrapartum death of nonmalformed fetuses in Denmark and Sweden in 1991. A perinatal audit. *Acta Obstet Gynecol Scand* 1997; **76**: 959–63.
39 Wilkinson D. Reducing perinatal mortality in developing countries. *Heal Policy Plan* 1997; **12**: 161–5.
40 Wolleswinkel-Van Den Bosch JH, Richardus J-H, Mackenbach JP, *et al.* Substandard factors in perinatal care in The Netherlands: A regional audit of perinatal deaths. *Acta Obstet Gynecol Scand* 2002; **81**: 17–24.
41 D'Aloja P, Salvatore MA, Sampaolo L, Privitera MG, Donati S, Group PMSSW. A nationwide surveillance system to reduce perinatal death cases in Italy: implementing a population-based pilot project. *Un Sist di sorveglianza Naz per la riduzione della mortalita Perinat Ital implementazione di un Progett Pilot Popul* 2021; **45**: 343–52.
42 Bandali S, Thomas C, Wamalwa P, *et al.* Strengthening the 'P' in Maternal and Perinatal Death Surveillance and Response in Bungoma county, Kenya: implications for scale-up. *BMC Health Serv Res* 2019; **19**: N.PAG-N.PAG.
43 Bausch LC, Smith C V. A focused review of fetal deaths in Nebraska in 1992. *Nebr Med J* 1996; **81**: 120–1.
44 Biswas A, Rahman F, Eriksson C, Halim A, Dalal K. Facility death review of maternal and neonatal deaths in Bangladesh. *PLoS One* 2015; **10**: e0141902.
45 Chepkin S, Prince S, Johnston T, *et al.* Learning from standardised reviews when babies die. National perinatal mortality review tool: first annual report. Oxford: National Perinatal Epidemiology Unit, 2019.
46 Harper RG, Sokal MM, Sokal S, Mastrota VF, Davis JG. The high-risk perinatal registry. A systematic approach for reducing perinatal mortality. *Obstet Gynecol* 1977; **50**: 264–8.
47 Hinderaker SG, Olsen BE, Bergsjø PB, *et al.* Avoidable stillbirths and neonatal deaths in rural Tanzania. *BJOG An Int J Obstet Gynaecol* 2003; **110**: 616–23.
48 Hundley V. Determining success in the provision of maternity care. PQDT - UK Irel. 2001. https://www.proquest.com/dissertations-theses/determining-success-provision-maternity-care/docview/301575818/se-2?accountid=13042.
49 Kieltyka L, Craig M, Goodman D, Wise R. Louisiana Implementation of the National Fetal and Infant Mortality Review (NFIMR) Program Model: Successes and Opportunities. *Matern Child Heal J* 2012; **16**: 353–9.
50 Mo-suwan L, Isaranurug S, Chanvitan P, *et al.* Perinatal death pattern in the four districts of thailand: Findings from the Prospective Cohort Study of Thai Children (PCTC). *J Med Assoc Thail* 2009; **92**:

660–6.
51 PMMRC. Fourteenth Annual Report of the Perinatal and Maternal Mortality Review Committee | Te Pūrongo ā-Tau Tekau mā Whā o te Komiti Arotake Mate Pēpi, Mate Whaea Hoki: Reporting mortality and morbidity 2018 | Te tuku pūrongo mō te mate me te whakamate 2018. Wellington: Health Quality & Safety Commission., 2021.
52 Rhoda N, Greenfield D, Muller M, *et al.* Experiences with perinatal death reviews in South Africa-the Perinatal Problem Identification Programme: scaling up from programme to province to country. *BJOG An Int J Obstet Gynaecol* 2014; **121**: 160–6.
53 Sharma B, Aggarwal N, Suri V, Siwatch S, Kakkar N, Venkataseshan S. Facility-based stillbirth surveillance review and response: an initiative towards reducing stillbirths in a tertiary care hospital of India. *J Perinat Med* 2022. DOI:https://dx.doi.org/10.1515/jpm-2021-0440.
54 The National Center for Fatality Review and Prevention. Fetal and Infant Mortality Review Manual: A Guide for Communities. Okemos: National Center for Fatality Review and Prevention, 2021.
55 Wilkins A, Earnest J, Mccarthy EA, Shub A. A retrospective review of stillbirths at the national hospital in Timor-Leste. *Aust N Z J Obstet Gynaecol* 2015; **55**: 331–6.
56 Amaral E, Souza JP, Surita F, *et al.* A population-based surveillance study on severe acute maternal morbidity (near-miss) and adverse perinatal outcomes in Campinas, Brazil: the Vigimoma Project. *BMC Pregnancy Childbirth* 2011; **11**: 9.
57 Andersen K V, Lange AP, Helweg-Larsen K. A perinatal audit of stillbirths in three Danish counties. *Scand J Soc Med* 1991; **19**: 127–33.
58 Cross-Sudworth F, Williams M, Gardosi J. Perinatal deaths of migrant mothers: Adverse outcomes from unrecognised risks and substandard care factors. *Br J Midwifery* 2015; **23**: 734–40.
59 De la Puente ML, Marti A, Pascual E, *et al.* Analysis of perinatal mortality in Bages (Barcelona). *Rev Calid Asist* 2002; **17**: 106–10.
60 Dekker CJ, Springer MP, Kanhai HHH. Perinatal mortality in a region where obstetric primary care is provided by the general practitioner: An audit. *Huisarts Wet* 2003; **46**: 191–5.
61 Draper E, Kurinczuk J, Kenyon S, on behalf of MBRRACE-UK. MBRRACE-UK 2017 Perinatal Confidential Enquiry: Term, singleton, intrapartum stillbirth and intrapartum-related neonatal death. The Infant Mortality and Morbidity Studies, Department of Health Sciences, University of Leicester: Leicester, 2017.
62 Maternal and Child Health Research Consortium. Confidential Enquiry into Stillbirths and Deaths in Infancy: 6th annual report. London: Maternal and Child Health Research Consortium, 1999.
63 Mersey Region Working Party on Perinatal Mortality. Confidential inquiry into perinatal deaths in the Mersey region. *Lancet (London, England)* 1982; **1**: 491–4.
64 Richardus JH, Graafmans WC, Bergsjo P, *et al.* Suboptimal care and perinatal mortality in ten European regions: methodology and evaluation of an international audit. *J Matern Fetal Neonatal Med* 2003; **14**: 267–76.
65 Stratulat P, Curteanu A, Caraus T, Petrov V, Gardosi J. The experience of the implementation of perinatal audit in Moldova. *BJOG An Int J Obstet Gynaecol* 2014; **121**: 167–71.
66 Tan KH, Wyldes MP, Settatree R, Mitchell T. Confidential regional enquiry into mature stillbirths and neonatal deaths--a multi-disciplinary peer panel perspective of the perinatal care of 238 deaths. *Singapore Med J* 1999; **40**: 251–5.
67 Tang A-W, Sabir N, Comber K, Liebling R, Pollard J, Roberts D. A pro forma and review process for the assessment of standards of care in stillbirths. *BJOG* 2011; **118**: 1661–4.
68 Vallejo BC. Confidential inquiry into perinatal mortality in the Lower Rio Grande Valley, 1988. ProQuest Diss. Theses. 1991. https://www.proquest.com/dissertations-theses/confidential-inquiry-into-perinatal-mortality/docview/303943485/se-2?accountid=13042.
